# Auxin regulates bulbil initiation by mediating sucrose metabolism in *Lilium lancifolium*

**DOI:** 10.1093/hr/uhae054

**Published:** 2024-02-23

**Authors:** Yin Xin, Xi Chen, Jiahui Liang, Shaokun Wang, Wenqiang Pan, Jingxiang Wu, Mingfang Zhang, Michele Zaccai, Xiaonan Yu, Xiuhai Zhang, Jian Wu, Yunpeng Du

**Affiliations:** Ornamental & Edible Lily Engineering Research Center of National Forestry and Grassland, Institute of Grassland, Flowers and Ecology, Beijing Academy of Agriculture and Forestry Sciences, Beijing 100097, China; Beijing Key Laboratory of Development and Quality Control of Ornamental Crops, Department of Ornamental Horticulture and Landscape Architecture, China Agricultural University, Beijing 100193, China; Ornamental & Edible Lily Engineering Research Center of National Forestry and Grassland, Institute of Grassland, Flowers and Ecology, Beijing Academy of Agriculture and Forestry Sciences, Beijing 100097, China; College of Landscape Architecture, Beijing Key Laboratory of Ornamental Plants Germplasm Innovation & Molecular Breeding, National Engineering Research Center for Floriculture, Beijing Laboratory of Urban and Rural Ecological Environment, Beijing Forestry University, Beijing 100083, China; Ornamental & Edible Lily Engineering Research Center of National Forestry and Grassland, Institute of Grassland, Flowers and Ecology, Beijing Academy of Agriculture and Forestry Sciences, Beijing 100097, China; Beijing Key Laboratory of Development and Quality Control of Ornamental Crops, Department of Ornamental Horticulture and Landscape Architecture, China Agricultural University, Beijing 100193, China; Beijing Key Laboratory of Development and Quality Control of Ornamental Crops, Department of Ornamental Horticulture and Landscape Architecture, China Agricultural University, Beijing 100193, China; Ornamental & Edible Lily Engineering Research Center of National Forestry and Grassland, Institute of Grassland, Flowers and Ecology, Beijing Academy of Agriculture and Forestry Sciences, Beijing 100097, China; Beijing Key Laboratory of Development and Quality Control of Ornamental Crops, Department of Ornamental Horticulture and Landscape Architecture, China Agricultural University, Beijing 100193, China; Ornamental & Edible Lily Engineering Research Center of National Forestry and Grassland, Institute of Grassland, Flowers and Ecology, Beijing Academy of Agriculture and Forestry Sciences, Beijing 100097, China; Department of Life Sciences, Ben-Gurion University of the Negev, Beer Sheva 8410501, Israel; College of Landscape Architecture, Beijing Key Laboratory of Ornamental Plants Germplasm Innovation & Molecular Breeding, National Engineering Research Center for Floriculture, Beijing Laboratory of Urban and Rural Ecological Environment, Beijing Forestry University, Beijing 100083, China; Ornamental & Edible Lily Engineering Research Center of National Forestry and Grassland, Institute of Grassland, Flowers and Ecology, Beijing Academy of Agriculture and Forestry Sciences, Beijing 100097, China; Beijing Key Laboratory of Development and Quality Control of Ornamental Crops, Department of Ornamental Horticulture and Landscape Architecture, China Agricultural University, Beijing 100193, China; Ornamental & Edible Lily Engineering Research Center of National Forestry and Grassland, Institute of Grassland, Flowers and Ecology, Beijing Academy of Agriculture and Forestry Sciences, Beijing 100097, China

## Abstract

Lily bulbils, which serve as advantageous axillary organs for vegetative propagation, have not been extensively studied in terms of the mechanism of bulbil initiation. The functions of auxin and sucrose metabolism have been implicated in axillary organ development, but their relationship in regulating bulbil initiation remains unclear. In this study, exogenous indole-3-acetic acid (IAA) treatment increased the endogenous auxin levels at leaf axils and significantly decreased bulbil number, whereas treatment with the auxin polar transport inhibitor *N*-1-naphthylphthalamic acid (NPA), which resulted in a low auxin concentration at leaf axils, stimulated bulbil initiation and increased bulbil number. A low level of auxin caused by NPA spraying or silencing of auxin biosynthesis genes *YUCCA FLAVIN MONOOXYGENASE-LIKE 6* (*LlYUC6*) and *TRYPTOPHAN AMINOTRANSFERASERELATED 1 *(*LlTAR1*) facilitated sucrose metabolism by activating the expression of *SUCROSE SYNTHASES 1* (*LlSusy1*) and *CELL WALL INVERTASE 2* (*LlCWIN2*), resulting in enhanced bulbil initiation. Silencing *LlSusy1* or *LlCWIN2* hindered bulbil initiation. Moreover, the transcription factor BASIC HELIX-LOOP-HELIX 35 (LlbHLH35) directly bound the promoter of *LlSusy1*, but not the promoter of *LlCWIN2*, and activated its transcription in response to the auxin content, bridging the gap between auxin and sucrose metabolism. In conclusion, our results reveal that an LlbHLH35-*LlSusy1* module mediates auxin-regulated sucrose metabolism during bulbil initiation.

## Introduction

*Lilium lancifolium*, a bulbous plant belonging to the family Liliaceae, is widely distributed in China and exhibits remarkable environmental adaptability [1]. This plant species serves both medicinal and culinary purposes, as its bulbs are rich in starch, saponin, colchicine, and other bioactive compounds. Lily bulbils, which are small bulbs generated from leaf axils, are rare vegetative reproductive organs in plants, playing a crucial role in ensuring plant survival, especially in natural triploid species like *L. lancifolium* [2]. At the onset of bulbil development, the first two layers of axil cells begin to differentiate into dividing cells, and the axillary meristem (AM) forms afterwards [3]. Consequently, bulbils and lateral branches can be considered homologous structures, sharing certain similarities in their initiation processes.

Auxin plays a vital role in AM initiation and lateral organ formation [4]. AM originates from a cell cluster maintaining *SHOOT MERISTEMLESS* (*STM*) expression and then develops into various lateral organs [5]. A minimal amount of auxin in the leaf axil is necessary for *STM* expression and meristematic cell maintenance [6, 7], which aligns with the findings from the auxin signaling reporters [8]. Ectopically increased auxin concentration in leaf axils can inhibit *STM* expression and AM initiation [9]. Meanwhile, high expression of auxin response factor MONOPTEROS (MP) also suppresses lateral organ initiation [10]. The TARs–YUCs module mediates the canonical two-step auxin synthesis pathway, coordinating both branch initiation and subsequent branch outgrowth. Excessive auxin levels, resulting from *YUC*s overexpression, hinder *WUSCHEL* (*WUS*) transcription and therefore repress branch initiation [11–13]. In contrast, NPA, a key inhibitor of auxin directional transport, efficiently restricts basal polar auxin flow to leaf axils, thus promoting the development of lateral branches [14, 15]. Consequently, the dynamic regulation of auxin levels emerges as a crucial factor in regulating lateral branch formation. In *Dioscorea alata* L., the localization of auxin at leaf axils regulates bulbil initiation [16], similar to observations in lilies, where decapitated ‘Sorbonne’ plants develop aerial bulbils at lower stems [17]. Auxin also influences bulblet initiation *in vitro* on the basal part of bulb scales [18]. These findings underscore the instrumental role of auxin in both bulbil and bulblet formation. However, the precise impact of different levels of auxin on bulbil initiation remains unclear.

Sucrose metabolism is crucial for providing energy and essential intermediate products for plant growth [19]. Sucrose transport occurs through the phloem, where it is loaded at the source and unloaded at the sink [20]. Upon unloading, sucrose is degraded into uridine diphosphate glucose (UDPG) and fructose via the action of sucrose synthases (SUSs), or it can be hydrolyzed into glucose and fructose by sucrose invertase (IVR) [21, 22]. The activities of SUS and IVR contribute to maintaining an appropriate sucrose concentration. Increased expression of genes involved in sucrose metabolism, such as those encoding SUS and IVR pathways, can promote early organogenesis. In the species *Gladiolus hybrida* and *Lycoris*, specific genes like *GhSUS2* and *LsSUS4* facilitate the process of sucrose downloading, leading to an increase in the number of corms and bulblets [23, 24]. Furthermore, a class of IVR called cell wall invertase can regulate sucrose transport by the apoplast pathway and promote cell division. *Arabidopsis* genes *AtCWIN2* and *AtCWIN4* positively regulate ovule formation [25]. Although sucrose metabolism is important for organogenesis, there are limited reports about the regulation of bulbil initiation. Additionally, sugars derived from sucrose metabolism can function as plant signaling regulators, interacting with light, hormones, and other growth factors [26]. Sucrose can antagonize the inhibitory effects of strigolactone (SL) on rice tillering [27]. Glucose, which is a metabolite of sucrose, accelerates bud growth by reducing endogenous abscisic acid (ABA) [28]. Trehalose 6-phosphate (T6P) derived from glucose interacts with SNF1-related protein kinase-1 complex to regulate plant carbon–nitrogen nutrient signaling [29]. However, the precise mechanism by which auxin and sugars cooperate to regulate bulbil development remains elusive.

In this study, we found that a low concentration of auxin in leaf axils, induced by NPA treatment or silencing of *LlYUC6* and *LlTAR1*, enhanced sucrose metabolism by activating the expression of *LlSusy1* and *LlCWIN2*, resulting in facilitated bulbil initiation. Furthermore, we showed that a bHLH transcription factor, LlbHLH35 directly activated *LlSusy1* in response to auxin signaling, which provides a mechanistic link between auxin content and sucrose metabolism during bulbil initiation. Overall, our study proposes that manipulating auxin levels can be an effective strategy to enhance lily bulbil yields.

## Results

### Morphological and histological description of bulbil formation in *L. lancifolium*

**Figure 1:**
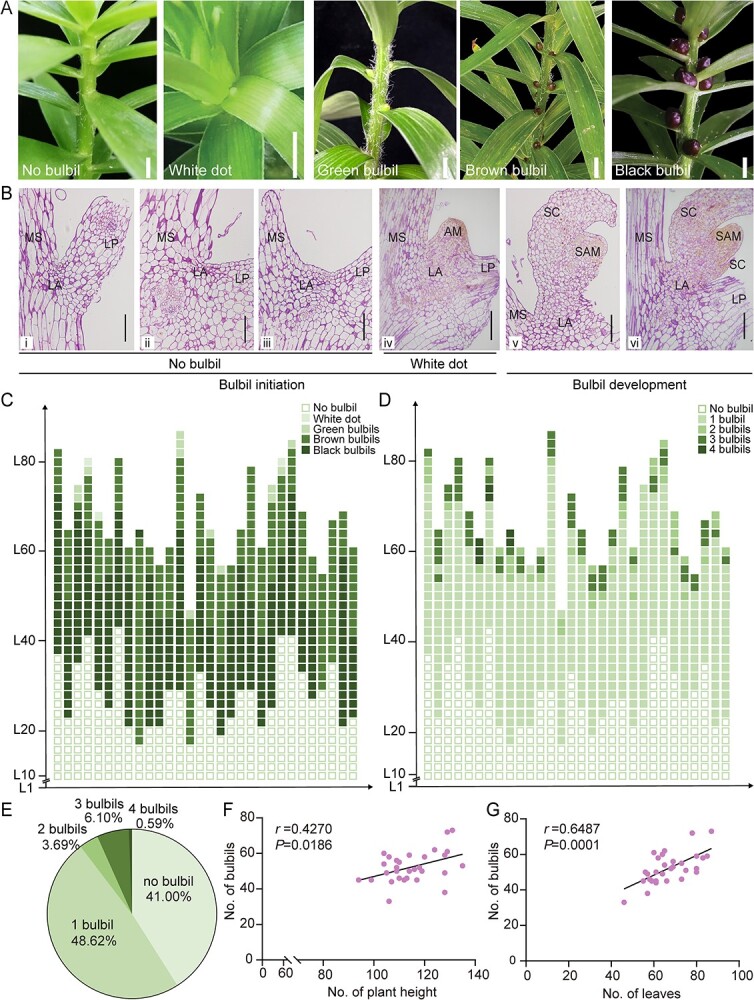
Morphological and histological observation of bulbil development. **A** Phenotypes of bulbil development in leaf axils from the first day of planting and continuing for 60 days. All white scale bars represent 1 cm. **B** Histological observations of bulbil formation. (i)–(iii) Epidermis cells of leaf axils underwent anticlinal and periclinal division, referred to as the ‘No bulbil’ stage. (iv) Apparent convexity appeared on the leaf axil, referred to as the ‘White dot’ stage. (v)–(vi) White bulbil dots further developed into mature bulbils with scales. All black scale bars represent 200 μm. AM, axillary meristem; LA, leaf axil; LP, leaf petiole; MS, main stem; SAM, shoot apical meristem; SC, scale. **C**, **D** Diagrammatic representation showing the distribution of bulbils in 30 independent plants on day 60 after planting: growth stages of bulbils (**C**) and number of bulbils per leaf axil (**D**). Each column represents a plant, and each box represents the type of bulbil. L1 represents the first leaf axil of the plant, and L means ‘leaf axil’. **E** Proportion of number of bulbils per leaf axil. Thirty biological replicates were performed. **F**, **G** Correlation between number of bulbils and plant height (**F**) and correlation between number of bulbils and number of leaves (**G**). Thirty biological replicates were performed. Pearson’s correlation (*r*) analyses (*P*<0.05) and scatter plots show the data distribution.

To understand bulbil formation in *L. lancifolium*, bulbil-related characters were continuously observed (Fig. 1A). Initially, no bulbils were observed in leaf axils. At ~20 days after planting, white bulbil dots appeared on the leaf base of the upper stem node. As the stem elongated, subsequent bulbils sequentially manifested in an upward direction. With the expansion, initial bulbils gradually changed from white to green, followed by browning with a tight scale structure. On day 60 after planting, the bulbils located along the lower section of the stem had matured to an almost black color, with scales tightly wrapping the internal shoot apical meristem (Fig. 1A). The bulbils had completed their growth stages 60 days after planting, and day 60 was chosen as the RNA-seq sampling point with treatments and bulbil phenotypes in gene-silenced plants in this study. Meanwhile, upper leaf axils lacking bulbils at 60 days after planting were sampled for additional analyses. Diagrammatic representations show the bulbil development pattern on whole plants (Fig. 1C and D). The black bulbils lower on the stem developed earlier than the upper ones, indicating that bulbils were maturing from bottom to top (Fig. 1C). The number of bulbils in each leaf axil was counted (Fig. 1D and E); 41.00% of leaf axils had no bulbils, 48.62% contained one bulbil, and ~10.38% contained multiple bulbils. Most leaf axils produced only one bulbil (Fig. 1E). Moreover, correlation analyses revealed that the number of bulbils was significantly positively correlated with plant height and number of leaves (Fig. 1F and G).

To gain further insights into bulbil formation, we conducted histological observations (Fig. 1B). During the ‘No bulbil’ stage, epidermis cells of leaf axils predominantly underwent anticlinal division in the tender tissues and then switched to periclinal division (Fig. 1Bi, ii). Accompanied by an enrichment of starch granules (purplish-red staining), the density of epidermis cells and the number of cell layers were increased (AM initiation), but without visible convexity (Fig. 1Biii). Subsequently, in the ‘White dot’ stage, apparent convexity or white bulbil dots occurred at the leaf axils with starch granules and proteins (yellow staining) (Fig. 1Biv). Finally, the bulging structure gradually generated scales (Fig. 1Bv, vi).

### Sucrose metabolism-mediated auxin regulates bulbil initiation

**Figure 2:**
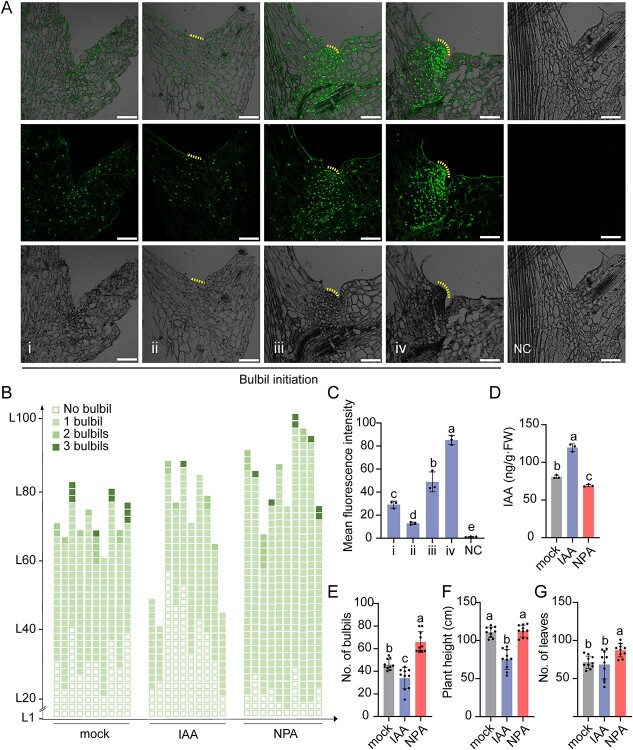
NPA and IAA regulate bulbil initiation by modulating auxin levels. **A** Immunolocalization of IAA in leaf axils at the ‘No bulbil’ stage corresponding to Fig. 1B. Auxin concentrations decreased in leaf axils (ii) prior to AM initiation (iii). Immunofluorescence assays were performed using an anti-IAA monoclonal antibody to investigate auxin distribution. Negative controls (NC) consisted of specimens that were not incubated with the anti-auxin antibody. Yellow lines represent AM initiation. Scale bars represent 200 μm. **B** Diagrammatic representation showing the position and number of bulbils per leaf axil in the mock, 100 mg/L IAA, and 100 mg/L NPA treatments on day 60 after planting. Ten independent plants were used for each treatment. Each column represents one plant, and each box represents the type of bulbil with different numbers of bulbils. L1 represents the first leaf axil, and L means ‘leaf axil’. Mock treatments using water with DMSO served as controls. **C** Mean fluorescence intensity of auxin in leaf axils. **D** Endogenous auxin contents in leaf axils with mock, IAA, and NPA treatments. Three biological replicates were performed in panels **C** and **D**. **E–G** Number of bulbils per plant (**E**), plant height (**F**), and number of leaves (**G**) in each plant. Ten biological replicates were performed. Lowercase letters in panels **C**–**G** represent significant differences calculated by ANOVA and *post hoc* Tukey’s HSD (*P* < 0.05).

Previous studies reported that auxin plays an essential role in AM and lateral organ initiation [30]. To investigate the role of auxin in bulbil production, we first detected the distribution and concentration of auxin by IAA immunolocalization assay. The lowest immunofluorescence signal in leaf axils occurred prior to AM initiation, suggesting that a low-auxin environment may be critical for bulbil initiation (Fig. 2Aii, iii and C). Next, plants 10 cm in height were treated with IAA and NPA (Fig. 2B). At day 60 after planting, compared with the control, IAA treatment reduced plant height and the number of bulbils, while NPA increased the number of leaves and bulbils and caused extremely curled leaves (Fig. 2E–G; Supplementary Data Fig. S1A and B). Moreover, NPA treatment resulted in decreased auxin in leaf axils, whereas spraying IAA supplied and increased auxin (Fig. 2D). The expression of **PIN-FORMED7* (*LlPIN7*)* involved in auxin polar transport was significantly downregulated in leaf axil regions by NPA [31] (Supplementary Data Fig. S1C). Additionally, axillary organogenesis genes *LlSTM* and *CUP-SHAPED COTYLEDON2* (*LlCUC2*) [30] were expressed highly in leaf axils after NPA treatment (Supplementary Data Fig. S1D and E). These results suggested that a lower auxin content caused by NPA facilitates the initiation of bulbils (as special axillary organs).

**Figure 3:**
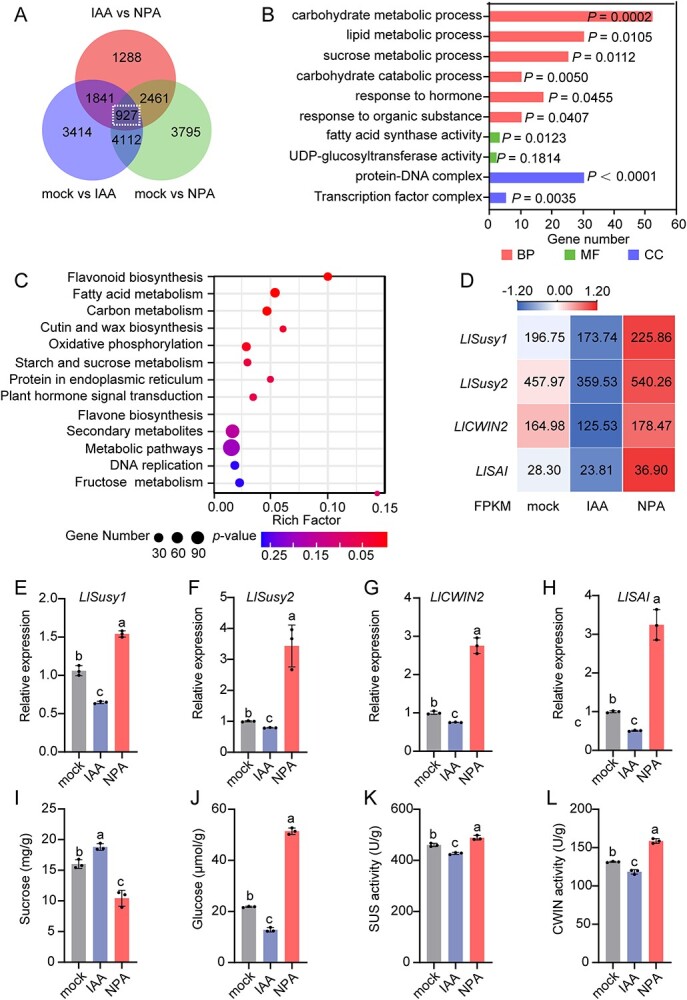
NPA and IAA control sucrose metabolism. **A** Venn diagram showing the number of DEGs in IAA versus NPA, mock versus IAA, and mock versus NPA comparisons. **B**, **C** Significantly enriched GO (**B**) and KEGG (**C**) pathways of 927 DEGs. Dot color means the *P*-value, and dot size represents the gene number enriched in the corresponding pathway. BP, biological process; MF, molecular function; CC, cellular component. **D** Heat map showing expression patterns of four sucrose-related DEGs in leaf axils with the mock, IAA, and NPA treatments. The color scale from blue to red represents the FPKM value from low to high. **E**–**H** Relative expression of *LlSusy1* (**E**), *LlSusy2* (**F**), *LlCWIN2* (**G**), and *LlSAI* (**H**) in leaf axils with the mock, IAA, and NPA treatments, verified by RT–qPCR. **I**–**L** Content of sucrose (**I**) and glucose (**J**), and enzyme activities of SUS (**K**) and CWIN (**L**) in leaf axils with the mock, IAA, and NPA treatments. Three biological replicates were performed for panels **E**–**L** and lowercase letters represent significant differences calculated by ANOVA and *post hoc* Tukey’s HSD (*P* < 0.05).

To explore the regulatory mechanisms of bulbil initiation by auxin, we conducted transcriptome analysis on the mock-, IAA-, and NPA-treated groups. High-quality RNA-seq data (Q20 ≥ 92.65%, Q30 ≥ 86.45%) confirmed the reliability of the analysis (Fig. 3; Supplementary Data Table S2). Volcano maps revealed a total of 5936 upregulated and 4358 downregulated differentially expressed genes (DEGs) in the mock versus IAA comparison, and 6420 upregulated and 4875 downregulated DEGs in the mock versus NPA comparison (Supplementary Data Fig. S2A and B). Among these DEGs, 927 were differentially expressed in all treatment groups (Fig. 3A). Gene Ontology (GO) enrichment and Kyoto Encyclopedia of Genes and Genomes (KEGG) pathway enrichment analyses for these 927 DEGs revealed that terms associated with sucrose metabolism were significantly enriched (Fig. 3B and C).

**Figure 4:**
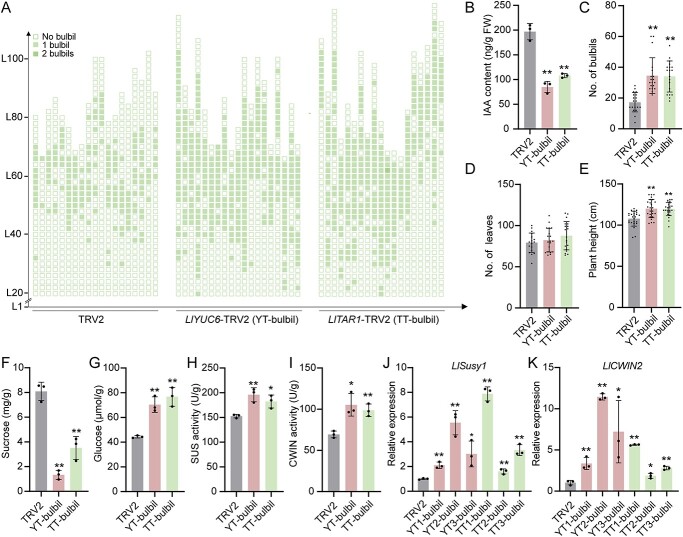
*LlYUC6* and *LlTAR1* negatively regulate lily bulbil initiation. **A** Diagrammatic representation showing the position and number of bulbils per leaf axil 60 days after planting. Nineteen independent TRV2, *LlYUC6*-TRV2 (YT-bulbils), or *LlTAR1*-TRV2 (TT-bulbils) plants were used for calculation. Each column represents one plant and each box represents the type of bulbil with different numbers of bulbils. L1 represents the first leaf axil of the plant and L means ‘leaf axil’. **B** Endogenous auxin contents in the leaf axils of TRV2, YT-bulbil, and TT-bulbil plants. Three biological replicates were performed. **C**–**E** Number of bulbils (**C**), number of leaves (**D**), and plant height (**E**) of each plant. Data represent mean ± standard deviation of 19 biological replicates. **F**–**I** Content of sucrose (**F**) and glucose (**G**) and activity of enzymes SUS (**H**) and CWIN (**I**) in leaf axils of TRV2, YT-bulbil, and TT-bulbil plants. **J**–**K** Relative expression of *LlSusy1* (**J**) and *LlCWIN2* (**K**) in leaf axils of TRV2, YT-bulbil, and TT-bulbil plants, verified by RT–qPCR. Data in panels **B** and **F**–**K** represent mean ± standard deviation of three biological replicates. Student’s *t*-test was used for statistical analysis (^*^P < 0.05; ^**^P < 0.01).

**Figure 5:**
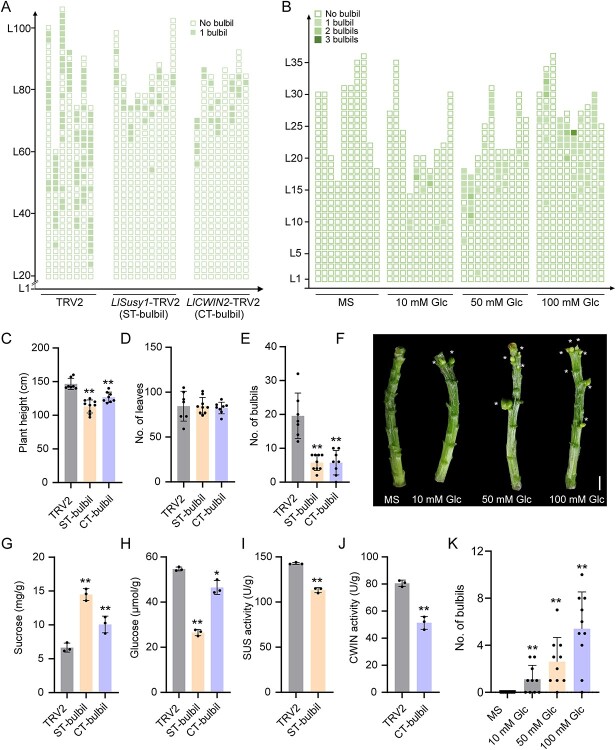
*LlSusy1* and *LlCWIN2* positively regulate lily bulbil initiation. **A** Diagrammatic representation showing the position and number of bulbils per leaf axil in silenced plants 60 days after planting. Seven independent TRV2 plants, nine *LlSusy1*-TRV2 (ST-bulbil) plants, or eight *LlCWIN2*-TRV2 (CT-bulbil) plants were used for calculation. Each column represents one plant, and each box represents the type of bulbil with different numbers of bulbils. L1 represents the first leaf axil of the plant and L means ‘leaf axil’. **B** Diagrammatic representation showing the position and number of bulbils per leaf axil of glucose treatment. Ten independent lily stem segments were grown on MS media containing 0, 10, 50, or 100 mM glucose (Glc). Each column represents one stem segment, and each box represents the type of bulbil with different numbers of bulbils. L means ‘leaf axil’. **C**–**E** Plant height (**C**), number of leaves (**D**), and number of bulbils (**E**) in each plant in the VIGS assays. Seven to nine independent plants were tested. **F** Phenotypes of stem segments grown on MS media with/without glucose after 16-day cultivation. White asterisks represent bulbils in leaf axils. The scale bar is 1 cm. **G**–**H** Content of sucrose (**G**) and glucose (**H**) in TRV2, ST-bulbil, and CT-bulbil plants. **I** Activity of SUS enzyme in leaf axils of TRV2 and ST-bulbil plants. **J** Activity of CWIN enzyme in leaf axils of TRV2 and CT-bulbil plants. Three biological replicates were performed in panels **G**–**J**. **K** Number of bulbils per stem segment grown on MS medium with/without glucose after 16-day cultivation. Ten biological replicates were performed. Data in panels **C**–**E** and **G**–**K** represent mean ± standard deviation. Student’s *t*-test was used for statistical analysis (^*^P < 0.05; ^**^P < 0.01).

It has been reported that SUS and IVR mediate sucrose metabolism in plants [24]. According to the reverse transcription–quantitative polymerase chain reaction (RT–qPCR), NPA treatment significantly increased the expression of genes involved in sucrose metabolism in leaf axils, including *LlSusy1*, *LlSusy2*, *LlCWIN2*, and *SOLUBLE ACID INVERTASE* (*LlSAI*), which was consistent with the FPKM (fragments per kilobase of exon model per million mapped fragments) value in the transcriptome. In contrast, IAA treatment reduced the expression of these genes (Fig. 3D–H). However, *LlSusyA*, *LlCWIN1*, *LlCWIN3*, and *LlCWIN4* did not have the opposite expression pattern in IAA and NPA treatments (Supplementary Data Fig. S2C). Moreover, *LlSusy1* and *LlCWIN2* were highly expressed at the ‘No bulbil’ stage while *LlSusy2* and *LlSAI* were highly expressed at the ‘White dot’ stage (Supplementary Data Fig. S2D), suggesting that *LlSusy1* and *LlCWIN2* may primarily degrade sucrose into UDPG at the bulbil initiation stage, while *LlSusy2* and *LlSAI* may function during bulbil growth.

To further investigate the role of auxin in sucrose metabolism, we examined the activities of SUS and CWIN, as well as the sucrose and glucose contents. NPA significantly increased the enzyme activities and glucose content, consistent with the decrease in sucrose content. Conversely, IAA treatment showed the opposite pattern, reducing enzyme activities and glucose content (Fig. 3I–L). These results indicate that the distinct auxin concentrations in leaf axils induced by NPA and IAA treatments may regulate bulbil initiation by modulating sucrose metabolism.

### Auxin biosynthesis genes *LlYUC6* and *LlTAR1* inhibit lily bulbil initiation

To further address the role of auxin in bulbil initiation, we utilized virus-induced gene silencing (VIGS) to silence two critical auxin biosynthesis-related genes—*LlYUC6* and *LlTAR1*—in *L. lancifolium* bulbs. Compared with the TRV2 control, silencing *LlYUC6* or *LlTAR1* resulted in decreased IAA content in leaf axils, leading to more bulbils (Fig. 4A–C; Supplementary Data Fig. S3A–D). In addition, silencing *LlYUC6* or *LlTAR1* increased plant height but did not change the number of leaves (Fig. 4D and E). Moreover, in these silenced plants, SUS activities, CWIN activities, and glucose content were increased, while sucrose content was decreased in leaf axils (Fig. 4F–I). In accordance with these findings, the expression of sugar-related genes (*LlSusy1* and *LlCWIN2*) and meristem-related genes (*LlSTM* and *LlCUC2*) was upregulated in leaf axils (Fig. 4J and K; Supplementary Data Fig. S3E and F). These changes resembled the effects of the NPA treatment, suggesting that maintaining an appropriate and lower content of auxin in leaf axils—either through restricted biosynthesis (*LlYUC6* or *LlTAR1* silencing) or NPA treatment—promotes bulbil initiation by enhancing sucrose metabolism.

### *LlSusy1* and *LlCWIN2* positively regulate lily bulbil initiation

To investigate the roles of *LlSusy1* and *LlCWIN2* in lily bulbil initiation, we silenced *LlSusy1* or *LlCWIN2* in bulbs using VIGS. Compared with the TRV2 control, we observed a decrease in bulbil formation of *LlSusy1*- and *LlCWIN2*-TRV2 plants, accompanied by lower plant height and no difference in the number of leaves (Fig. 5A and C–E; Supplementary Data Fig. S4A–D). Additionally, transcription levels of *LlSTM* and *LlCUC2* in leaf axils were downregulated (Supplementary Data Fig. S4E and F). These results suggest that both *LlSusy1* and *LlCWIN2* act as positive regulators of lily bulbil initiation.

Silencing *LlSusy1* or *LlCWIN2* resulted in increased sucrose and reduced glucose contents compared with the TRV2 control (Fig. 5G and H). Moreover, the SUS enzyme activity in *LlSusy1*-TRV2 plants and CWIN enzyme activity in *LlCWIN2*-TRV2 plants were decreased compared with those in the TRV2 control plants (Fig. 5I and J). To analyze the role of glucose during bulbil formation, we treated lily stem segments with exogenous glucose. After 16 days of treatment, segments with glucose developed bulbils at the leaf axils, and the highest number of bulbils was produced with the highest glucose treatment (100 mM), demonstrating that glucose promotes bulbil formation (Fig. 5B, F, and K).

Collectively, these results indicate that sucrose metabolism genes *LlSusy1* and *LlCWIN2* participate in regulating the sucrose-to-glucose process and promoting bulbil initiation in leaf axils.

### *LlYUC6*, *LlTAR1*, *LlSusy1*, and *LlCWIN2* participate in lily bulblet initiation

**Figure 6:**
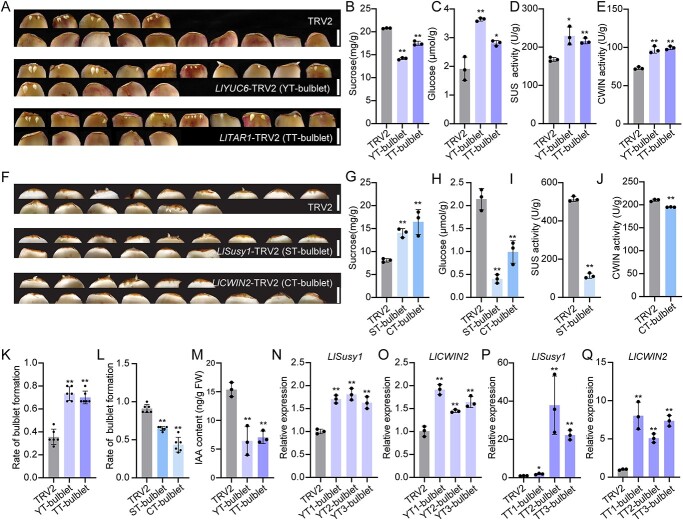
Silencing *LlYUC6*, *LlTAR1*, *LlSusy1*, and *LlCWIN2* alters lily bulblet initiation. **A** Phenotypes of bulblet initiation on *LlYUC6*-TRV2 (YT-bulblet), *LlTAR1*-TRV2 (TT-bulblet), and TRV2 scales after 3-week cultivation on water agar media (7 g/L). Scale bars represent 1 cm. **B**–**E** Content of sucrose (**B**) and glucose (**C**) and activity of enzymes SUS (**D**) and CWIN (**E**) were analyzed in the scale base of TRV2, YT-bulblet, and TT-bulblet plants. **F** Phenotypes of bulblet formation on *LlSusy1*-TRV2 (ST-bulblet), *LlCWIN2*-TRV2 (CT-bulblet), and TRV2 scales after 3-week cultivation on water agar media (7 g/L). Scale bars represent 1 cm. **G**, **H** Content of sucrose (**G**) and glucose (**H**) in the scale base of TRV2, ST-bulblet, and CT-bulblet. **I** Activity of SUS enzyme in the scale base of TRV2 and ST-bulblet. **J** Activity of CWIN enzyme in the scale base of TRV2 and CT-bulblet. **K** Rates of bulblet formation in YT-bulblet, TT-bulblet, and TRV2 lines. **L** Rates of bulblet formation in ST-bulblet, CT-bulblet, and TRV2 lines. Rates of bulblet formation in panels **K**–**L** were determined as the ratio of the number of scales with bulblets to total number of scales. Six biological replicates (*n* = 15 scales per replicate) were performed. **M** Endogenous auxin contents in YT-bulblet, TT-bulblet, and TRV2 lines. Three biological replicates were performed in panels **B**–**E**, **G**–**J**, and **M**. **N**, **O** Relative expression analysis of *LlSusy1* (**N**), and *LlCWIN2* (**O**) in scale bases of YT-bulblet and TRV2 lines. **P, Q** Relative expression analysis of *LlSusy1* (**P**) and *LlCWIN2* (**Q**) in scale bases of TT-bulblet and TRV2 lines. Expression in panels **N**–**Q** was detected by RT–qPCR and data represent mean ± standard deviation of three biological replicates. Student’s *t*-test was used for statistical analysis in panels **B**–**E** and **G**–**Q** (^*^P < 0.05; ^**^P < 0.01).

Lily bulbils, also referred to as ‘aerial bulblets,’ share similarities with bulblets formed by lily scales. As a stable genetic system has not been established in *L. lancifolium*, we determined the roles of *LlYUC6*, *LlTAR1*, *LlSusy1*, and *LlCWIN2* in bulbil initiation by silencing these four genes in *L. lancifolium* scales and then observing the rate of bulblet formation (Fig. 6; Supplementary Data Fig. S5). In the base part of scales, (i) compared with the TRV2 control, *LlYUC6*- and *LlTAR1*-silenced scales exhibited increased rates of bulblet formation, with lower levels of IAA and sucrose (Fig. 6A B, K, and M), increased glucose content and activity of the enzymes SUS and CWIN (Fig. 6C–E), and upregulated expression of *LlSusy1*, *LlCWIN2*, *LlSTM*, and *LlCUC2* (Fig. 6N–Q; Supplementary Data Fig. S5A, B, and E–J); (ii) silencing *LlSusy1* or *LlCWIN2* decreased the rate of bulblet formation and glucose content but increased sucrose content (Fig. 6F–H); and (iii) SUS activity in *LlSusy1*-silenced scales, CWIN activity in *LlCWIN2*-silenced scales, and expression levels of *LlSTM* and *LlCUC2* were significantly lower than those in the TRV2 control (Fig. 6I and J; Supplementary Data Fig. S5C, D, and K–P). These results mirrored the effects observed in *LlYUC6-*, *LlTAR1-*, *LlSusy1-*, and *LlCWIN2-*silenced bulbils (Figs 4 and 5).

**Figure 7:**
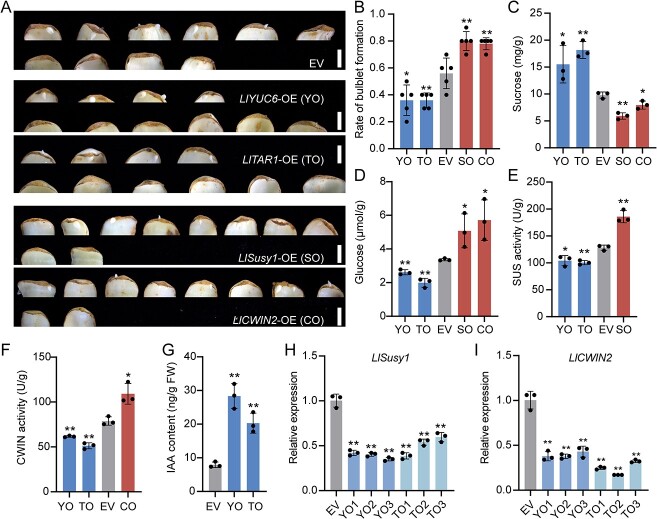
Overexpression of *LlYUC6*, *LlTAR1*, *LlSusy1*, and *LlCWIN2* control lily bulblet initiation. **A** Phenotypes of bulblet initiation on *LlYUC6*-OE (35S:eGFP-*LlYUC6*; YO), *LlTAR1*-OE (35S:eGFP-*LlTAR1*; TO), *LlSusy1*-OE (35S:eGFP-*LlSusy1*; SO), *LlCWIN2*-OE (35S:eGFP-*LlCWIN2*; CO), and EV (35S:eGFP) scales after 3-week cultivation on water agar media (7 g/L). Scale bars represent 1 cm. **B** Rates of bulblet formation in YO, TO, SO, CO, and EV lines. The rate of bulblet formation was determined by the ratio of number of scales with bulblets to total number of scales. Five biological replicates were performed (*n* = 10 scales per replicate). **C**, **D** Contents of sucrose (**C**) and glucose (**D**) in the scale base of YO, TO, SO, CO, and EV lines. **E** Activity of SUS enzyme in the scale base of YO, TO, SO, and EV lines. **F** Activity of CWIN enzyme in YO, TO, CO, and EV lines. **G** Endogenous auxin contents in YO, TO, and EV lines. **H**, **I** Relative expression analysis of *LlSusy1* (**H**) and *LlCWIN2* (**I**) in the scale base of YO, TO, and EV lines, detected by RT–qPCR. Data in panels **C**–**I** represent mean ± standard deviation of three biological replicates. Student’s *t*-test (^*^P < 0.05; ^**^P < 0.01).

To further explore the roles of *LlYUC6*, *LlTAR1*, *LlSusy1*, and *LlCWIN2* in bulblet initiation, we transiently overexpressed these four genes in *L. lancifolium* scales by using agroinfiltration (Fig. 7; Supplementary Data Fig. S6). In the base part of the scales, overexpression of *LlYUC6* and *LlTAR1* resulted in decreased rates of bulblet formation with increased levels of IAA and sucrose compared with the control; glucose content, SUS and CWIN activities, and expression of *LlSusy1*, *LlCWIN2*, *LlSTM*, and *LlCUC2* were all downregulated (Fig. 7A–I; Supplementary Data Fig. S6A, B, and E–H). Conversely, the rate of bulblet formation, glucose content, and the expression of *LlSTM* and *LlCUC2* in the base tissues of scales were significantly upregulated in *LlSusy1*- and *LlCWIN2*-overexpressing scales, with a reduction in sucrose content (Fig. 7A–D; Supplementary Data Fig. S6C, D, and I–L). Additionally, SUS activity in *LlSusy1*-overexpressing scales and CWIN activity in *LlCWIN2*-overexpressing scales were both increased compared with that of the empty vector (EV) (Fig. 7E and F). These findings were opposite to the effects observed in *LlYUC6*-, *LlTAR1*-, *LlSusy1*-, and *LlCWIN2-*silenced scales (Fig. 6).

Collectively, our results revealed that maintaining an appropriate and lower content of auxin in the base part of scales is crucial for bulblet initiation, and that auxin biosynthesis genes *LlYUC6* and *LlTAR1* negatively regulate bulblet initiation. Moreover, sucrose metabolic genes *LlSusy1* and *LlCWIN2* positively regulate bulblet initiation, consistent with their role in bulbil initiation.

### LlbHLH35 directly activates *LlSusy1* expression in response to auxin signaling

**Figure 8:**
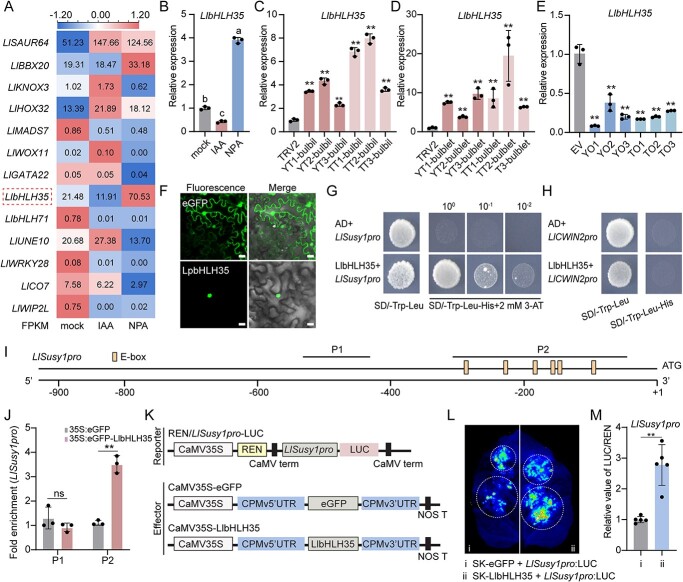
LlbHLH35 directly activates *LlSusy1* expression in response to auxin signaling. **A** Expression profile of 13 potential transcription factors in response to auxin signaling selected from the transcriptome database. The color scale from blue to red represents the FPKM value from low to high. **B**–**E** Relative expression analysis of *LlbHLH35* in leaf axils of mock, IAA, and NPA treatments (**B**), leaf axils of *LlYUC6*-TRV2-bulbil (YT-bulbil), *LlTAR1*-TRV2-bulbil (TT-bulbil), and TRV2 bulbil lines (**C**), scale base tissues of *LlYUC6*-TRV2-bulblet (YT-bulblet), *LlTAR1*-TRV2-bulblet (TT-bulblet), and TRV2 lines (**D**), and the scale base of *LlYUC6*-OE-bulblet (YO), *LlTAR1*-OE-bulblet (TO), and EV (empty vector) bulblet lines (**E**). Data represent mean ± standard deviation of three biological replicates. **F** LlbHLH35 protein was fused with an eGFP tag, and empty vector (35S:eGFP) was used as a control. The scale bar is 20 μm. **G**, **H** Interaction between LlbHLH35 and the *LlSusy1* promoter (*LlSusy1pro*) (**G**) or *LlCWIN2* promoter (*LlCWIN2pro*) (**H**) was determined in yeast one-hybrid assays by yeast growth on synthetic dropout (SD) nutrient media lacking Trp, Leu, and His with 2 mM (**G**) or without (**H**) 3-amino-1,2,4-triazole (3-AT). The same results were obtained in at least five independent yeast cells. **I** Schematic diagram of *LlSusy1* genomic regions upstream of the start codon. Yellow boxes represents E-box elements. Thin black lines mark the fragments used for the ChIP–qPCR assay. P1, −535 bp to −431 bp; P2, −324 bp to −55 bp. **J** ChIP–qPCR analyses of binding of LlbHLH35 to *LlSusy1pro*. Embryonic calluses expressing 35S:eGFP-LlbHLH35 were used for ChIP. 35S:eGFP was used as the internal control. P1 of *LlSusy1pro* was used as negative control for binding of LlbHLH35. Antibodies against the eGFP tag were used. Data represent mean ± standard deviation of three biological replicates (*n* = 6 calluses per replicate). **K**–**M** LlbHLH35 activation of the *LlSusy1* promoter. Schematic diagram of dual-LUC vectors (**K**). *LlSusy1pro* was fused to LUC reporter and LlbHLH35 was used as the effector (eGFP was the negative control). Images of firefly luciferase in the *N. benthamiana* leaf were taken on the third day after agroinfiltration (**L**). The same results were obtained in six biological replicates. Quantitative analysis of luciferase activity (**M**). The combination of eGFP and *LlSusy1pro* was used for normalization (set to 1). Data represent mean ± standard deviation of six biological replicates (*n* = 4 leaf discs per replicate). Different letters in panel **B** indicate significant differences by ANOVA Turkey’s HSD tests for pairwise comparisons (*P* < 0.05). Student’s *t*-test was used for statistical analysis in panels **C**–**E**, **J**, and **M** (^**^P < 0.01; ns, not significant).

Given that transcription factors (TFs) are widely involved in hormone signaling, sugar metabolism, organ development, and other processes [32], we reanalyzed the 927 DEGs and identified 13 TFs (Figs 3A and 8A). Examination of the expression pattern of these 13 TFs with their FPKMs revealed one bHLH family member, *LlbHLH35* (Supplementary Data Fig. S7), that exhibited the highest expression in the NPA group, but the lowest expression in the IAA group. This suggested that *LlbHLH35* may play a role in response to auxin signaling and regulating bulbil initiation (Fig. 8A).

Subsequently, we validated the expression pattern of *LlbHLH35* by RT–qPCR and found that *LlbHLH35* was induced by NPA and repressed by IAA, consistent with its FPKM values (Fig. 8A and B). Moreover, compared with expression in the TRV control, *LlbHLH35* was upregulated in *LlYUC6*- or *LlTAR1*-silenced bulbils/bulblets and downregulated in *LlYUC6*- or *LlTAR1*-overexpressing scales (Fig. 8C–E). These observations suggest that auxin inhibits *LlbHLH35* expression.

To further characterize the function of LlbHLH35 and bridge the gaps between auxin signaling and sucrose metabolism, we first monitored its subcellular localization and found that LlbHLH35 was localized in the nucleus in *Nicotiana benthamiana* epidermal cells (Fig. 8F). Then, we investigated whether auxin-responsive LlbHLH35 directly regulates *LlSusy1* and *LlCWIN2*. Given that bHLHs bind the G-box element (CACGTG) and more generally the E-box (CANNTG) [33], we found that promoters of *LlSusy1* (*LlSusy1pro*) and *LlCWIN2* (*LlCWIN2pro*) contained several E-boxes (Fig. 8I; Supplementary Data Fig. S8). Using a yeast one-hybrid assay, LlbHLH35 was able to bind *LlSusy1pro* but not *LlCWIN2pro* (Fig. 8G and H). This interaction between LlbHLH35 and *LlSusy1pro* was confirmed using a chromatin immunoprecipitation–quantitative PCR (ChIP–qPCR) assay, with LlbHLH35 binding the P2 region containing E-boxes (Fig. 8I and J). Subsequently, dual-luciferase reporter assays (dual-LUC) in *N. benthamiana* were employed to validate the effect of LlbHLH35 on *LlSusy1pro*. Leaves co-infiltrated with SK-LlbHLH35 (effector) and *LlSusy1pro*-LUC (reporter) exhibited significantly higher LUC activity compared with leaves infiltrated with SK and *LlSusy1pro*-LUC, suggesting that LlbHLH35 activates *LlSusy1* expression (Fig. 8K–M)*.*

Taken together, these results show that *LlbHLH35* is repressed by auxin and positively regulates *LlSusy1* by directly binding E-boxes of the *LlSusy1* promoter.

### *LlbHLH35* positively regulates lily bulblet initiation

**Figure 9:**
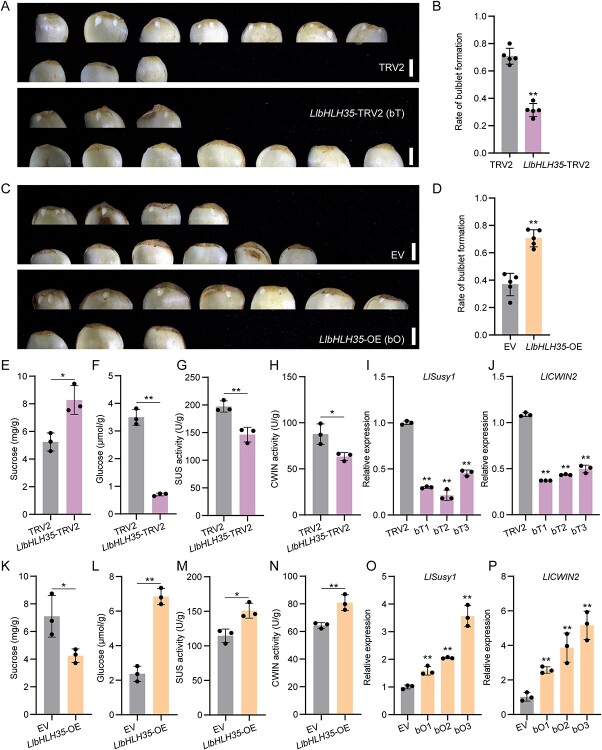
*LlbHLH35* positively regulates lily bulblet initiation. **A** Phenotypes of bulblet initiation on *LlbHLH35*-TRV2 (bT) and TRV2 scales after 3-week cultivation on water agar media (7 g/L). Scale bars represent 1 cm. **B** Rates of bulblet formation in *LlbHLH35-*TRV2 and TRV2 lines. **C** Phenotypes of *LlbHLH35-*OE (35S:eGFP-*LlbHLH35*; bO) and EV (empty vector) scales. Scale bars represent 1 cm. **D** Rates of bulblet formation in *LlbHLH35-*OE and EV lines. In panels **A**–**D** five biological replicates (10 scales per replicate) were performed. The rate of bulblet formation was determined as the ratio of number of scales with bulblets to total number of scales. **E**–**H** Contents of sucrose (**E**) and glucose (**F**) and activities of enzymes SUS (**G**) and CWIN (**H**) in *LlbHLH35-*TRV2 and TRV2 lines. **I**, **J** Relative expression of *LlSusy1* (**I**) and *LlCWIN2* (**J**) in scale bases of *LlbHLH35-*TRV2 and TRV2 lines, verified by RT-qPCR. **K**–**N** Contents of sucrose (**K**) and glucose (**L**) and activities of enzymes of SUS (**M**) and CWIN (**N**) in *LlbHLH35-*OE and EV lines. **O**, **P** Relative expression of *LlSusy1* (**O**) and *LlCWIN2* (**P**) in scale bases of *LlbHLH35-*OE (bO) and EV lines, verified by RT–qPCR. Data in panels **E**–**P** represent mean ± standard deviation of three biological replicates. Student’s *t*-test was used for statistical analysis in panels **B**, **D**, and **E**–**P** (^*^P < 0.05; ^**^P < 0.01).

To explore the role of *LlbHLH35* in lily bulbil and bulblet initiation, we silenced this gene by VIGS in *L. lancifolium* scales and observed the rate of bulblet formation. Compared with the TRV2 control, silencing *LlbHLH35* decreased the rate of bulblet formation, glucose content, and activities of SUS and CWIN, but increased the sucrose content (Fig. 9A, B, and E–H). Moreover, *LlSusy1*, *LlCWIN2*, *LlSTM*, and *LlCUC2* were downregulated in the base of *LlbHLH35-*silenced scales (Fig. 9I and J; Supplementary Data Fig. S9A and C–E).

To further validate the role of *LlbHLH35* in bulblet initiation, we transiently overexpressed *LlbHLH35* (*LlbHLH35*-OE) in *L. lancifolium* scales. Compared with the control, *LlbHLH35*-OE scales had an increased rate of bulblet formation (Fig. 9C and D). In addition, the sucrose content was reduced, but the glucose content, SUS and CWIN activities, and the expression of *LlSusy1*, *LlCWIN2, LlSTM*, and *LlCUC2* were upregulated in the base of *LlbHLH35*-OE scales (Fig. 9K–P; Supplementary Data Fig. S9B and F–H), contrary to the results in *LlbHLH35*-silenced scales.

Collectively, these results suggest that *LlbHLH35* positively regulates lily bulblet initiation by inducing the sucrose metabolic gene *LlSusy1*.

## Discussion

**Figure 10:**
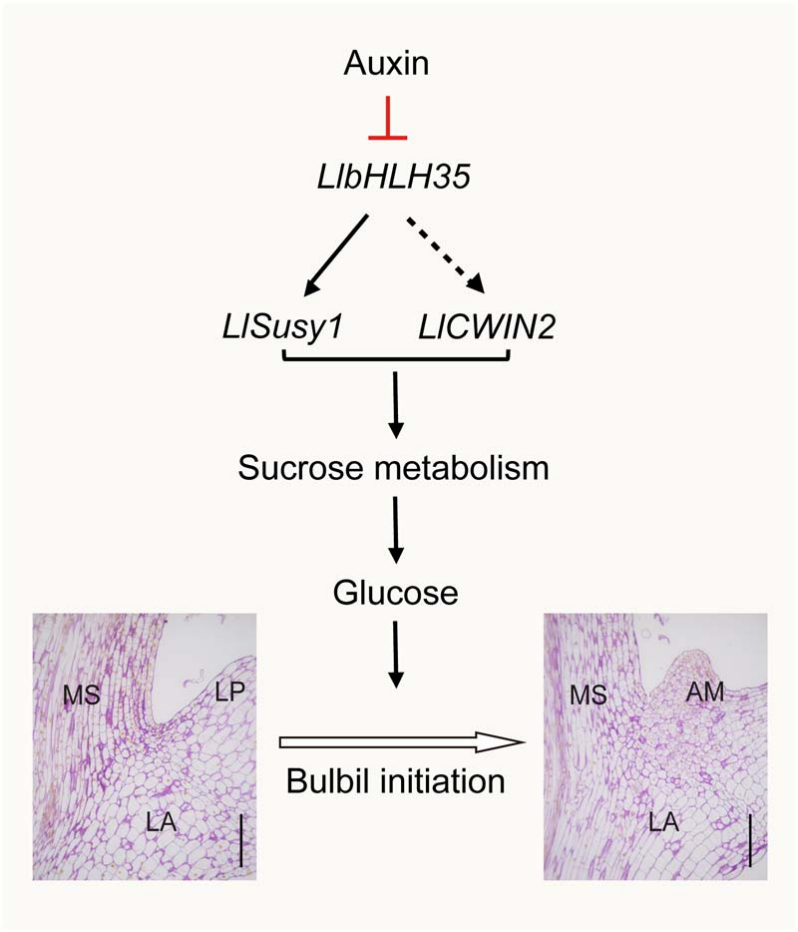
A model of auxin and sucrose metabolism in bulbil initiation in *L. lancifolium*. During bulbil initiation, a low level of auxin in leaf axils is required to activate the expression of *LlbHLH35*, which upregulates sucrose-related genes *LlSusy1* and *LlCWIN2*, along with the enzyme activities of SUS and CWIN. This process leads to an increase in endogenous glucose content in leaf axils, contributing to bulbil initiation. AM, axillary meristem; LA, leaf axil; LP, leaf petiole; MS, main stem. Scale bars represent 200 μm.

The bulbil, as a specific axillary organ, plays a vital role in vegetative reproduction in lilies. Increasing bulbil production is highly advantageous for lily propagation [1]. Auxin, which is a key regulatory plant hormone, controls the development of various axillary organs, including branches, spines, and runners [34]. Removing the apical bud or externally applying NPA promotes the growth of axillary buds and other tissues, the phenomenon known as ‘release of apical dominance’ [35, 36]. On the other hand, sucrose metabolism, specifically the conversion of sucrose to glucose, is closely associated with the development of AMs [37]. However, how auxin and sucrose cooperatively control bulbil formation in lilies has yet to be elucidated. In this study, exogenous IAA treatment at a high concentration did not promote bulbil formation, whereas NPA treatment inhibited auxin accumulation in leaf axils and increased the number of bulbils. Further investigations in bulbils and bulblets revealed that a suitable, low concentration of auxin (caused by NPA treatment or silencing of *LlYUC6* and *LlTAR1*) promoted sucrose metabolism by positively regulating *LlSusy1* and *LlCWIN2*, thereby increasing bulbil and bulblet initiation. Moreover, LlbHLH35 activated *LlSusy1* expression in response to auxin signaling, bridging the gap between auxin content and sucrose metabolism (Fig. 10).

### A low concentration of auxin benefits lily bulbil and bulblet initiation

Auxin is an essential signaling molecule that regulates various stages of plant growth [38]. Ectopic production of auxin by an auxin biosynthetic gene, *iaaM*, in leaf axils interferes with AM formation, whereas repression of auxin signaling in polar auxin transport mutants can largely rescue their branching defects [39]. Thus, a local minimum (but indispensable) level of auxin is critical for the upregulation of *STM*, as well as for the formation of WUS–CLAVATA3 (CLV3) modules, which are necessary for the initiation of axillary buds [30]. Meanwhile, *STM* forms a feedback regulation loop with *CUC*s to regulate AM initiation [30]. However, the role of auxin in regulating lily bulbil initiation is unclear.

NPA functions by competing for the auxin-binding site of the PIN transporter, inhibiting the conformational change of PIN, and subsequently blocking the polar transport of auxin [40]. In horticultural practice, exogenous NPA spraying and decapitation of the terminal bud are commonly employed to remove apical dominance, stimulate lateral branching, and promote fruit production [14, 41]. In addition, moderating auxin biosynthesis can regulate lateral organ initiation. The production of auxin primarily relies on tryptophan (TRP)-dependent biosynthetic pathways, where enzymes TAR and YUC are essential [42]. Studies on moss gametophores through *tarac* and *yuccf* mutants showed that inhibiting *TAR* and *YUC* genes can increase lateral branching and reduce apical dominance [13]. In this study, IAA immunolocalization assay revealed that the upper leaf axils maintained a low level of auxin throughout the stem elongation process, with a relatively low auxin level before bulbil initiation, indicating a critical role for a low-auxin environment in bulbil initiation (Fig. 2A). Exogenous NPA treatments increased the yield of bulbils with a decreased auxin content in leaf axils and caused extremely curled leaves (Fig. 2; Supplementary Data Fig. S1A and B), suggesting that NPA inhibited auxin polar transport in the main stem and leaves to mediate bulbil initiation and leaf patterning. Furthermore, silencing of *LlYUC6* or *LlTAR1*—both of which are auxin biosynthesis-related genes—in lily bulbs resulted in increased bulbils with a reduced IAA concentration in leaf axils, similar to the effects observed with NPA treatments (Fig. 4). Moreover, the expression of *LlSTM* and *LlCUC2* was upregulated by NPA treatments and the silencing of *LlYUC6* or *LlTAR1* (Supplementary Data Figs S1D and E and S3E and F). These results support the idea that decreased levels of auxin in leaf axils promote lily bulbil initiation.

The initiation of bulbils and bulblets produced by scales shares a common process in lilies. Previous studies have shown that after the epidermis cells of the scale base undergo vigorous cell division with abundant protein and starch, bulblets form as small white dots, similar to bulbil initiation [43, 44]. Here, we used *L. lancifolium* scales for VIGS to investigate the rate of bulblet formation (Fig. 6). Compared with TRV2 lines, silencing of *LlYUC6* or *LlTAR1* led to an increased rate of bulblet initiation while exhibiting a lower auxin content and the downregulation of *LlSTM* and *LlCUC2* (Fig. 6; Supplementary Data Fig. S5G–J). Our previous study demonstrated that cytokinins promote bulblet formation on scales, while simultaneously reducing endogenous auxin levels [45]. These results are consistent with those in bulbil initiation, suggesting that a conserved mechanism exists in lily bulbil and bulblet initiation. However, further research is required to understand the fine-tuned regulation of bulbil and bulblet initiation.

### The auxin–sucrose module is a critical mediator for organ formation

In sucrose metabolism, the enzymes SUS and IVR are instrumental in unloading sucrose from the phloem in ‘sink’ tissues. This process provides essential nutrients and signaling molecules that are involved in organ formation and plant growth [26, 46]. Examples of such processes include the feedback loop (*CINV1*–glucose level–auxin–*HXK1*–miR156–*SPL9*–*PAP1*–*CINV1*–glucose) promoting juvenile-to-adult transition and branch formation in *Arabidopsis*, and the involvement of CWIN and vacuolar invertase (VIN) in sucrose-induced stem branching in potato [47, 48]. Moreover, sucrose metabolism can cooperate with auxin signaling to facilitate cell division and expansion during organ formation. For instance, auxin/IAA inhibits the activity of SUS during tomato fruit ripening, and *AtCWIN*s are directly regulated by ARF TFs and participate in *Arabidopsis* flower development [49, 50]. Additionally, in rice, auxin regulates sucrose partitioning from flag leaves (source) to reproductive tissues (sink) [51]. Our study revealed an auxin–sucrose module that regulates bulbil initiation: a low concentration of auxin, induced by NPA treatment or silencing of *LlYUC6* and *LlTAR1*, promoted sucrose degradation by upregulating expression of *LlSusy1* and *LlCWIN2* and enzyme activities of SUS and CWIN, which positively regulated bulbil initiation (Figs 2, 4, and 5). We obtained the same conclusion for bulblet initiation (Figs 6 and 7). Therefore, our findings suggest that a low concentration of auxin regulates lily bulbil initiation by affecting sugar metabolism.

Glucose derived from sucrose degradation is transformed into T6P under catalysis by hexokinase (HXK) and trehalose-6-phosphate synthase (TPS), and the concentration of T6P contributes to bud outgrowth and branching by inhibiting the protein kinases KIN10 and KIN11 [29]. In our study, we observed that T6P content and the expression of T6P-related genes (*LlHXK2*, *LlTPS1*, *LlTPS6*, and *LlKIN10*) were increased by NPA treatment, but decreased by IAA treatment (Supplementary Data Fig. S10). These findings suggest that the auxin–sucrose module also influences T6P concentrations. Consequently, the role of sucrose metabolite T6P in lily bulbil initiation is intriguing and warrants further investigation.

### *LlbHLH35* mediates sucrose metabolism in response to auxin signaling

The bHLH TFs are widely involved in plant stress responses and light and hormone signaling [33]. In the auxin signaling pathway, the bHLH protein PHYTOCHROME INTERACTING FACTOR4 (PIF4) activates auxin response genes to regulate thermoresponsive growth in *Arabidopsis* [52]. The IAA–LEUCINE RESISTANT 3 (*Rh*ILR3; *Rh*bHLH105)–LESION SIMULATING DISEASE ONE LIKE 1 (*Rh*LOL1) module regulates petal abscission by directly binding to the RhIAA4-1 promoter in roses [53]. Additionally, MdbHLH3 regulates sugar metabolism in apples by binding directly to the promoter of *PYROPHOSPHATE-DEPENDENT PHOSPHOFRUCTOKINASE* (*MdPFPβ*) to activate its expression, thereby increasing fructose-6-phosphate and sucrose concentrations [54]. In rice, OsbHLH067, OsbHLH068, and OsbHLH069 redundantly regulate inflorescence AM formation [55]. However, whether bHLHs integrate auxin and sugar signaling to regulate organ formation remains unclear. In this study, we discovered that LlbHLH35 is involved in the auxin response and sucrose metabolism. *LlbHLH35* was upregulated by a decreased auxin concentration, activating the sucrose-degrading gene *LlSusy1* and promoting bulbil and bulblet initiation. Conversely, *LlbHLH35* was downregulated by exogenous IAA treatment or overexpression of *LlYUC6* and *LlTAR1*, which further inhibited sucrose metabolism and bulbil and bulblet initiation (Figs 8 and 9). Moreover, although both overexpression and silencing of *LlbHLH35* affected *LlCWIN2* expression and CWIN enzyme activity, LlbHLH35 could not directly bind to the *LlCWIN2* promoter (Figs 8 and 9). These findings suggest that LlbHLH35 may regulate *LlCWIN2* expression in an indirect manner. Further investigations are needed to explore these mechanisms.

## Materials and methods

### Plant materials and treatments

Bulbs of *L. lancifolium*, free from diseases and with dormancy broken, were collected from the National Lily Germplasm Bank at Beijing Academy of Agricultural and Forestry Sciences (BAAFS). The diameter of these bulbs ranged from 3.5 cm to 5.0 cm. All bulbs were soaked in diluted carbendazim solution (1:500 dilution) for 30 min, washed with clean water, and planted in pots in the greenhouse at BAAFS, with conditions comprising a constant average temperature of 24.5 ± 2.5°C, a 16/8 h light/dark cycle, and relative humidity of 40–60%.

To observe bulbil development, 30 plants were randomly selected. On day 60 after planting, plant height was measured and the numbers of leaves and bulbils were counted on each plant. The growth stage of bulbils in leaf axils was recorded and the number of bulbils per leaf axil on each plant was determined. Pearson’s correlation analysis, conducted via GraphPad Prism (version 9), examined the relationships between bulbil numbers and plant height as well as leaf numbers.

For exogenous hormone treatments, IAA or NPA was dissolved in dimethyl sulfoxide (DMSO) as the stock solution. Working solutions containing 100 mg/L IAA or 100 mg/L NPA were sprayed every 3 days, starting from the 10-cm height stage, and continuing until the visible flower stage (60 days after planting). Mock treatment with DMSO was used as control. A total of 10 independent plants were used in each treatment. On day 60 after planting, plant height was measured and the numbers of leaves and bulbils were counted on each plant. Upper leaf axil-portion tissues with no bulbil were cut for RT–qPCR and metabolite analysis. The leaf rolling index (LRI, %) was calculated by measuring the length of fully expanded rolled leaves (Ll), measuring the length of normal leaves (Ln), and then using the formula: (Ll − Ln) /Ll × 100%. Three leaves were randomly selected and measured from each plant to obtain an average value.

### Histological analysis

The first tissues of the leaf axil on the upper stem node were sampled ~20 days after planting, located above the lower nodes with a white bulbil dot. During the process of stem elongation, leaf axils with the original and developed bulbils were sequentially sampled for histological observation. Tissues were cut and fixed with 50% formaldehyde–acetic acid–ethanol (FAA) buffer for >24 h at room temperature. Then, tissues were completely dehydrated with an alcohol gradient of 75, 85, 90, 95%, and anhydrous ethanol. After alcohol benzene and xylene treatments, the transparent tissues were soaked in melted paraffin at 65°C for 1 day. The wax-soaked tissues were processed using a paraffin slicer to obtain 5-μm-thick sections. Then, paraffin sections were stained with periodic acid–Schiff (PAS) dye solution, rinsed with naphtholsulfone S and dried using anhydrous ethanol. Starch granules and cell walls were stained purplish red, and proteins were stained yellow.

### Virus-induced gene silencing in bulbils and bulblets

A specific fragment (350 bp) of each target gene (*LlYUC6*, *LlTAR1*, *LlSusy1*, and *LlCWIN2*) and a 300-bp sequence of *LlbHLH35* were cloned into a pTRV2 vector. The constructs pTRV1, pTRV2, and pTRV2-specific fragment were transformed into *Agrobacterium* strain EHA105. Silencing of the target genes by VIGS in bulbils or bulblets was performed as described previously [56].

For VIGS in bulbils, *L. lancifolium* bulbs with buds of 4–5 cm were selected. Bulbs were submerged in infiltration buffers containing the pTRV2-specific fragment and pTRV1, or pTRV2 and pTRV1, then were planted in a moist medium mixture of peat and vermiculite (1:1, v/v) at 23 ± 2°C under 16/8 h light/dark conditions and relative humidity of 40–60%. On day 60 after planting, plant height, number of leaves, and number of bulbils produced per plant were counted. Upper leaf axils without bulbils were sampled for gene expression analysis and measurement of auxin and physiological parameters. At least eight individual plants (bulbs) were used for each gene-silencing vector.

For VIGS in bulblets, middle scales from 6- to 8-cm circumference lily bulbs without disease were selected. After infiltration, the scales were cultivated on water agar media (7 g/L) and placed in a completely dark incubator (23 ± 2°C). After 3 weeks, the rate of bulblet formation was determined as the ratio of the number of scales with bulblets to the total number of scales, and the bases of the scales (bulblet removed) were excised for further analysis. Experiments were repeated three times, with six biological replicates (15 scales per replicate) in each repetition for *LlYUC6-*, *LlTAR1-*, *LlSusy1-*, and *LlCWIN2-*silenced lines and with five biological replicates (10 scales per replicate) for *LlbHLH35*-silenced lines.

### Transient gene overexpression in bulblets

The open reading frame (ORF) region for each of the genes *LlYUC6*, *LlTAR1*, *LlSusy1*, *LlCWIN2*, and *LlbHLH35* was individually cloned into the 35S:*eGFP*/pCAMBIA2300 vector. The resulting vectors with EV were transformed individually into *Agrobacterium* strain EHA105 for transient gene overexpression in scales. Bacteria were grown to an optical density at 600 nm (OD_600_) of 1.0 and were resuspended in resuspension buffer (10 mM MgCl_2_, 10 mM MES, pH 5.8, 200 μM acetosyringone). Middle-layer scales from 6- to 8-cm-circumference lily bulbs without disease were selected and then infiltrated with the resuspension buffer. Infiltration was performed under vacuum (0.8 MPa; 30 min), after which the scales were placed on water agar media (7 g/L) in a completely dark incubator (23 ± 2°C). After 3 weeks, the rate of bulblet formation was determined as the ratio of the number of scales with bulblets to the total number of scales, and the bases of scales (bulblet removed) were excised for further analysis. Experiments were repeated three times, with five biological replicates (10 scales per replicate) in each repetition.

### Measurement of endogenous auxin

Upper leaf axils without bulbils of plants under mock, IAA, and NPA treatments, and of *LlYUC6*- or *LlTAR1*-silenced plants on day 60 after planting were sampled for endogenous auxin measurement. In addition, the bases of *LlYUC6*- or *LlTAR1*-silenced scales and *LlYUC6*- or *LlTAR1*-overexpressing scales were sampled 3 weeks after infiltration.

Approximately 0.2 g of each sample was rapidly frozen in liquid nitrogen and homogenized into a powder. Endogenous IAA was extracted according to Chen *et al*. [57]. Quantitative analysis of IAA was conducted by high-performance liquid chromatography (HPLC)–electrospray ionization tandem mass spectrometry (HPLC–ESI–MS/MS) [58]. The IAA content was determined using the external standard method and was expressed as nanograms per gram fresh weight. Three biological replications were performed.

### Immunolocalization of IAA

IAA distribution was examined in accordance with a previously described protocol [59] with minor modifications. The tissue sections were first incubated with a 1:100 (v/v) dilution of anti-IAA monoclonal antibody (Sigma–Aldrich, A0855) for 2 h at 37°C in the dark, followed by a 1:500 (v/v) dilution of DyLight™ 488-labeled anti-mouse IgG antibody (KPL, 5230-0391) for 1 h at 37°C in the dark. Fluorescence signals were captured using a laser scanning confocal microscope (Nikon Eclipse Ti). Negative controls consisted of specimens that were not incubated with the anti-auxin antibody.

### Metabolite analysis

Upper leaf axil portions without bulbils from hormone treatments and VIGS (for bulbil initiation) on day 60 after planting and the base tissues of scales from VIGS and transient overexpression assays (for bulblet initiation) 3 weeks after infiltration were frozen in liquid nitrogen and stored at −80°C. Sucrose and glucose contents were measured using a Sucrose Content Assay Kit (BC2460, Solarbio, Beijing, China) and a Glucose Content Assay Kit (BC2500, Solarbio), respectively. Sucrose synthase (EC 2.4.1.13) and cell wall acid invertase (EC 3.2.1.26) activities were measured using a Sucrose Synthase Activity Assay Kit (BC4310, Solarbio) and a Cell Wall-Bound Acid Invertase Activity Assay Kit (BC4320, Solarbio), respectively. A 0.1-g sample was used in all detection assays, and three biological replicates were analyzed.

### Glucose treatment

Consistently growing *L. lancifolium* plants without bulbils were selected for the glucose treatment. Stem segments with shoot apical meristem, ~60 cm in length, were excised for explant sterilization (75% alcohol for 2 min, then 3% sodium dichloroisocyanurate for 30 min). The sterilized stem segments were then cultured in MS media supplemented with various concentrations of glucose (10, 50, and 100 mM), while MS media served as the control. Ten individual stem segments were employed for each treatment, and observations of leaf axils were conducted every 2 days, with counting taking place on day 16.

### RNA-seq library construction, sequencing, and analysis of differentially expressed genes

Upper leaf axil portions without bulbils of plants under mock, IAA, and NPA treatments were collected on day 60 after planting and used for RNA-seq analysis. Three biological replicates were analyzed. Total RNA of each sample was extracted using an RNAprep Pure Plant Plus Kit (DP441, Tiangen, Beijing, China). Library construction was performed according to the NEB normal library building method [60]. The 150-bp paired-end reads were generated on an Illumina NovaSeq platform (Novogene, Beijing, China). The National Center for Biotechnology Information Sequence Read Archive (NCBI SRA) accession number is PRJNA916842. The final clean reads were assembled for subsequent analysis, with the clustered sequence used as a reference.

Gene expression concentrations were assessed according to FPKM. DEGs were determined by the DESeq2 R package (1.20.0) [61] with the standard *P*-value <0.01 or absolute fold change >1. Functions of DEGs were analyzed in GO (http://geneontology.org/) and KEGG (https://www.kegg.jp/) by using KOBAS software [62].

### RT–qPCR

Total RNA from samples was extracted using a RNAprep Pure Plant Plus Kit (DP441, Tiangen). First-strand cDNA was then synthesized according to the manual of a PrimeScript RT Reagent Kit with gDNA Eraser (RR047, TaKaRa, Tokyo, Japan). Specific primers for RT–qPCR were designed by the GenScript tool (https://www.genscript.com), and the primer sequences are listed in Supplementary Data Table S1. *F-BOX FAMILY PROTEIN* (*FP*) was used as the internal reference gene [63]. RT–qPCR was conducted using TB Green^®^ Premix Ex Taq™ II (RR820A, TaKaRa) in a Bio-Rad CFX96™ Real-Time System (CA, USA). The 2^−ΔΔCt^ method was used to analyze RT–qPCR expression data according to Wu *et al*. [56]. All RT–qPCR assays were performed with three biological replicates.

### Subcellular localization assay

The *LlbHLH35* coding sequence was fused with eGFP on the 35S:pCAMBIA2300 vector. The fusion vectors and 35S:*eGFP*/pCAMBIA2300 (positive control) were transformed into *Agrobacterium* strain EHA105. Bacterial solutions were resuspended to an OD_600_ of 1.0 in resuspension buffer (10 mM MgCl_2_, 10 mM MES, pH 5.8, 200 μM acetosyringone). After 3 h in the dark, the mixed solution with the target *Agrobacterium* and RNA silencing suppressor p19 (P19) in a 1:1 (v/v) ratio was injected into *N. benthamiana* leaves. After 3 days of infiltration, the injected leaves were observed for eGFP fluorescence signals using a Zeiss LSM-710 laser confocal microscope (Germany). eGFP was excited at 488 nm, and its emission was detected using a 515–535 nm band-pass filter.

### Dual-luciferase reporter assay in *N. benthamiana*

For the transactivation assay of LlbHLH35 on the *LlSusy1* promoter (*LlSusy1pro*), the *LlbHLH35* coding sequence was cloned into pGreenII 62-SK vector as the effector (SK-LlbHLH35), and *LlSusy1pro* was cloned into pGreenII 0800-LUC as the reporter (*LlSusy1pro*:LUC). The combination of SK-empty and *LlSusy1pro*:LUC was used as the negative control. All vectors were transformed into *Agrobacterium* strain GV3101 harboring the pSoup and p19 plasmids. Infiltration and luciferase (LUC) measurements were performed as previously described [64]. Six biological replicates were used in this assay.

### Chromatin immunoprecipitation–qPCR assay

ChIP–qPCR was performed as previously described with minor modifications [65]. Briefly, 35S:*eGFP*/pCAMBIA2300 (35S:eGFP) and 35S:*LlbHLH35*-eGFP/pCAMBIA2300 (35S:eGFP-LlbHLH35) vectors were transformed individually into *Agrobacterium* strain EHA105, and the bacterial solution was incubated to OD_600_ = 1.0, as described for the subcellular localization assay. Embryonic calluses of lilies were previously formed on medium containing 4.43 g/L MS, 1.0 mg/L picloram, 0.2 mg/L 1-naphthylacetic acid, 30 g/L sucrose, and 7 g/L agar. Then, lily calluses were submerged in the solution, placed under a vacuum (−25 kPa) for 15 min, and then cultivated on medium containing 4.43 g/L MS, 0.3 mg/L picloram, 0.1 mg/L 1-naphthylacetic acid, 200 μM acetosyringone, 30 g/L sucrose, and 7 g/L agar for 48 h in the dark. Subsequently, lily calluses were used for the ChIP–qPCR assay. The fold enrichment of promoters was analyzed using qPCR. Three biological replicates were used in this assay.

### Yeast one-hybrid assay

The *LlSusy1* promoter sequence (−362 bp to −55 bp; referred to as the transcription beginning) containing six E-box motifs or the *LlCWIN2* promoter sequence (−1279 bp to −867 bp; referred to as the start of transcription) containing four E-box motifs was cloned into pHIS2.1 as *LlSusy1pro*/pHIS2.1 or *LlCWIN2pro*/pHIS2.1. The resulting constructs were transformed into yeast strain Y187 and selected on SD/−Trp with 3-amino-1,2,4-triazole (3-AT) for the self-activation test. Next, the coding sequence of *LlbHLH35* was inserted into the pGADT7 vector to construct *LlbHLH35*/pGADT7. Subsequently, *LlbHLH35*/pGADT7 and *LlSusy1pro*/pHIS2.1, and *LlbHLH35*/pGADT7 and *LlCWIN2pro*/pHIS2.1 were co-transformed into yeast strain Y187 according to the Yeast Protocols Handbook (Clontech, USA). The pGADT7 empty plasmid with promoter/pHIS2.1 was used as a negative control. Successful transformants were selected by 3-AT on SD/−Trp−Leu−His at 30°C for 3 days.

### Statistical analysis

Data were statistically analyzed using one-way ANOVA with *post hoc* Tukey’s honestly significant difference (HSD) (*P* < 0.05) and Student’s *t*-test. GraphPad Prism (version 9) software was used for analyses.

## Acknowledgements

This work was supported by the National Natural Science Foundation of China (No. 32371954 and 32171864 to Y.D.; 32372740 and 32172617 to J.W.; 32302599 to J.L.), the Excellent Youth Science Foundation of Beijing Academy of Agriculture and Forestry Sciences (YXQN202303 to Y.D.), Pinduoduo-China Agricultural University Research Fund (PC2023B02009), Construction of Beijing Science and Technology Innovation and Service Capacity in Top Subjects (CEFF-PXM 2019_014207_000032), and the 2115 Talent Development Program of China Agricultural University, the Strategic Development Department of China Association for Science and Technology, 111 Project of the Ministry of Education (B17043), and China Postdoctoral Science Foundation (2023M740311).

## Author contributions

X.C. and Y.X. observed the development of bulbil and performed the endogenous hormone treatments; Y.X. and W.P. conducted the transcriptome analysis; X.C., Y.X., and S.W. performed gene silencing; Y.X., J.X.W., and M.F.Z. conducted the metabolite analysis; X.C., Y.X., J.L, J.W., and M.Z. wrote and rewrote the article; J.W., Y.D., X.Y., X.C., J.L., and X.Z. conceived the study and reviewed the article; all authors read and approved the article.

## Data availability statement

The raw sequence data of RNA-seq reported in this study have been deposited in the NCBI SRA data under accession number PRJNA916842. The authors declare that all data supporting the findings of this study are available from within the article and supplementary data or are available upon reasonable request from the corresponding author.

## Conflict of interest

The authors report no declarations of interest.

## Supplementary data

Supplementary data are available at *Horticulture Research* online.

## Supplementary Material

Web_Material_uhae054
