# Supplementary material for: Auxin regulates bulbil initiation by mediating sucrose metabolism in Lilium lancifolium
Source: Hortic Res. 2024 Feb 23;11(4):uhae054. doi: 10.1093/hr/uhae054 (PMC11069426; doi:10.1093/hr/uhae054)
Supplement: Web_Material_uhae054 [file web_material_uhae054.zip › Supplementary data.docx]

**Supplementary** **Table S1. Primers used in this study**

| **Primer name** | **Forward primer (5'-3')** | **Reverse primer (5'-3’)** |
| --- | --- | --- |
| **For Virus-induced gene silencing (VIGS)** | | |
| *LlYUC6*-TRV2-HR | agaaggcctccatggggatccGTCGGAGCCGGGCCTTCG | gggacatgcccgggcctcgagGCCAACGGGAAACATAGTCCC |
| *LlTAR1*-TRV2-HR | agaaggcctccatggggatccTACATTGTGGTGGGGACTGGC | gggacatgcccgggcctcgagAAAGAGCATTAGATCGTGATCGG |
| *LlSusy1-*TRV2 | GGAATTCGTTGCAACGCCATCAGTTG | GGGGTACCGCGTTGAAGGGCTCAAAGTC |
| *LlCWIN2-*TRV2 | GGAATTCTGGATCAATGATCCGAATGGC | GGGGTACCTCACGGAGAAACGGGTCT |
| *LlbHLH35*-TRV2-HR | gtgagtaaggttaccgaattcTAGCTTGATAAATGAGAAAAAACATATTTT | cgtgagctcggtaccggatccCTTGCAGCCCTTGTGAATTTTC |
| CP (Coat  Protein) | CCTGCTGACTTGATG  GACGA | GCCAGTGTTCGCCTTG  GTAGT |
| **For gene overexpression and protein subcellular localization** | | |
| *LlYUC6*-eGFP-HR | gccgaattccccgggggatccATGGACTGTTGGAGGGAGATGG | agggcatgcctgcaggtcgacTCACAATGCCGTCGGAGG |
| *LlTAR1*-eGFP-HR | gccgaattccccgggggatccATGGCGCCCTTTTCTCGC | agggcatgcctgcaggtcgacTCATGAGATAGATGATAATCTTTCAGTAAAA |
| *LlSusy1*-eGFP-HR | gccgaattccccgggggatccATGCCCAACCGCAGGTTG | agggcatgcctgcaggtcgacTTAGTTGTCAATTGCCAAGGGA |
| *LlCWIN2*-eGFP-HR | gccgaattccccgggggatccATGGAGCTCTCAAAGCTCACCA | agggcatgcctgcaggtcgacTTATAATCCATTCATGAGTGGTTTCTT |
| *LlbHLH35*-eGFP-HR | gccgaattccccgggggatccATGGATGACATTGATGCTGATTTC | agggcatgcctgcaggtcgacTCAATTTGTTTGTCGGAGTGAGAG |
| *eGFP-*test | CCATCTTCTTCAAGGACGACG | TCGATGTTGTGGCGGATCT |
| **For yeast one-hybrid (Y1H) system** | | |
| *LlSusy1pro*-pHIS | GGAATTCGGTTCATCTTTCTTCAATGGTC | GACTAGTGCCTTACAAGATCCCAAACTC |
| *LlCWIN2pro*-pHIS | GGAATTCAGGGTCAAGTCATGAGCAC | GACTAGTTCTTTCAGAAGTACGCAGCG |
| *LlbHLH35-*AD-HR | gccatggaggccagtgaattcATGGATGACATTGATGCTGATTTC | cagctcgagctcgatggatccTCAATTTGTTTGTCGGAGTGAGAG |
| **For dual-luciferase (Dual-LUC) reporter analysis** | | |
| *LlSusy1pro*-LUC-HR | ctatagggcgaattgggtaccAAGACCTTTGAGGCACGCCA | agaactagtggatcccccgggTCAACTTCCTTCAAGCCTTACAAG |
| *LlbHLH35-*SK-HR | cgctctagaactagtggatccATGGATGACATTGATGCTGATTTC | gataagcttgatatcgaattcTCAATTTGTTTGTCGGAGTGAGAG |
| **For Chromatin immunoprecipitation (ChIP) assay** | | |
| *LlSusy1pro*-ChIP-P1 | TGTTTCAAGGGTGGAACACCAGT | GCCTTACAAGATCCCAAACTCTGC |
| *LlSusy1pro*-ChIP-P2 | AGAGATCATTGGTGGCTTCGGT | GCCAATAATTGAGAAAGGCAAAGGGA |
| **For real-time quantitative PCR (RT-qPCR) analysis** | | |
| *LlSusy1*-qPCR | CATGGCGAGGCTGGACAGAG | GGCCACCACCACAAGGTTGA |
| *LlSusy2*-qPCR | CGGCCGACTCGGTCATCTTC | TGTGGGCACGGAGAAAGTCG |
| *LlCWIN2*-qPCR | CCGTTTCTCCGTGAGTGGCT | CCGTCGGATCCCATCCAAGC |
| *LlSAI*-qPCR | TCCTCCCTGGTGTCCTCCAC | TTAGCACATGCCGCACCGAT |
| *LlSTM*-qPCR | CTCTCCTCCTCCGCCCTCAC | CACGTGACGCCGGATCTCTC |
| *LlCUC2*-qPCR | CTCCGGGTTTCCGGTTCCAC | TTGCCCGCCATGGAAGATCC |
| *LlYUC6*-qPCR | GGCTCCCGGTAAAGGTCGTG | GCTCGAGCGGACCTAACTCG |
| *LlTAR1*-qPCR | TCCAAGTCCGCTCCATCCGA | GGCGCGATCTCCCATCCTTT |
| *LlPIN7*-qPCR | TCTGCCAATGCTCGGAGAGA | GCTGAGCAGGTAGCGATGAC |
| *LlbHLH35*-qPCR | CGGGTCAACGACTCGTCCTC | TCCTCCTCTCCTGCTCTTGGA |
| *LlHXK2*-qPCR | GGCGAAGAAGGCGGTGATCT | CACCGATCCTGCAGCTCCTT |
| *LlTPS1*-qPCR | CCGGTGTCGAGGGTGTTTGG | CCACGAATCCTCGCCCTTCC |
| *LlTPS6-*qPCR | CGCCAGCTCAAGGACACCAT | GCAGGGACGCAGTTGAAGGT |
| *LlKIN10-*qPCR | GCATTGGTTCCTTTGGCAAGGT | CGCCTCACTTTCTCTTCCATGTCC |
| *FP*-qPCR | TCGCCTACATCGCTAACC | TTCCCAATAATCGCAAGACC |
| HR: Homologous Recombination | | |

**Supplementary** **Table S2. Summary of transcriptome database**

| **Sample** | **Raw Reads/kb** | **Clean Reads/kb** | **Clean Bases/Gb** | **Error(%)** | **Q20(%)** | **Q30(%)** | **GC Content(%)** |
| --- | --- | --- | --- | --- | --- | --- | --- |
| mock-1 | 22872933 | 22722954 | 6.82 | 0.02 | 95.20 | 88.98 | 49.43 |
| mock-2 | 26292277 | 25619200 | 7.69 | 0.02 | 95.67 | 90.19 | 50.75 |
| mock-3 | 30895353 | 30032943 | 9.01 | 0.02 | 96.20 | 91.15 | 50.23 |
| IAA-1 | 31681238 | 31065268 | 9.32 | 0.02 | 95.71 | 90.15 | 51.68 |
| IAA-2 | 29270868 | 28467788 | 8.54 | 0.02 | 92.65 | 86.45 | 51.67 |
| IAA-3 | 25781477 | 25017211 | 7.51 | 0.02 | 96.20 | 91.15 | 51.18 |
| NPA-1 | 37981869 | 36204088 | 10.86 | 0.02 | 96.13 | 91.02 | 51.03 |
| NPA-2 | 17724040 | 17044927 | 5.11 | 0.02 | 93.75 | 87.95 | 51.20 |
| NPA-3 | 31842456 | 30976676 | 9.29 | 0.02 | 96.21 | 91.21 | 50.70 |
| Total | 254342511 | 247151055 | 74.15 |  |  |  |  |
| Average | 28260279 | 27461228.33 | 8.23889 | 0.02 | 95.302 | 89.8056 | 50.8744 |

**
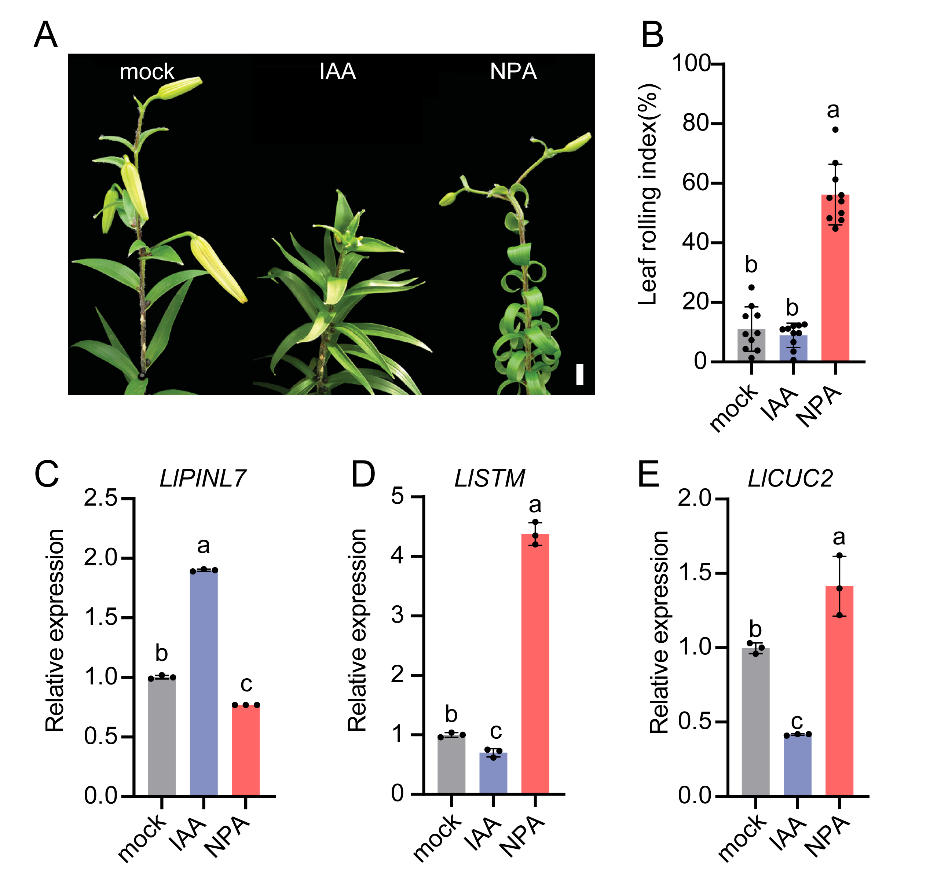
**

**Supplementary Figure S1. The effects of IAA and NPA treatment on *Lilium lancifolium*. (A)** The phenotypes of mock, IAA, and NPA treatment plants. 10 independent plants were used for each treatment. Bar represents 1 cm. **(B)** The leaf rolling index (LRI, %) in the mock, IAA, and NPA treatments. LRI (%) = (Ll-Ln)/Ll×100%. Ll represents the maximum leaf length; Ln represents the leaf length in the natural state. Ten biological replicates (three leaves per plant) were performed. **(C)-(E)** Relative expression analysis of *LlPIN7* **(C)**, *LlSTM* **(D)**, and *LlCUC2* **(E)** in leaf axils with mock, IAA, and NPA treatment verified by RT-qPCR. The data represents mean ± s.d. of three biological replicates. The lowercase letters in panels **B-E** represent significant differences calculated by an ANOVA and post hoc Tukey’s HSD (P＜0.05).


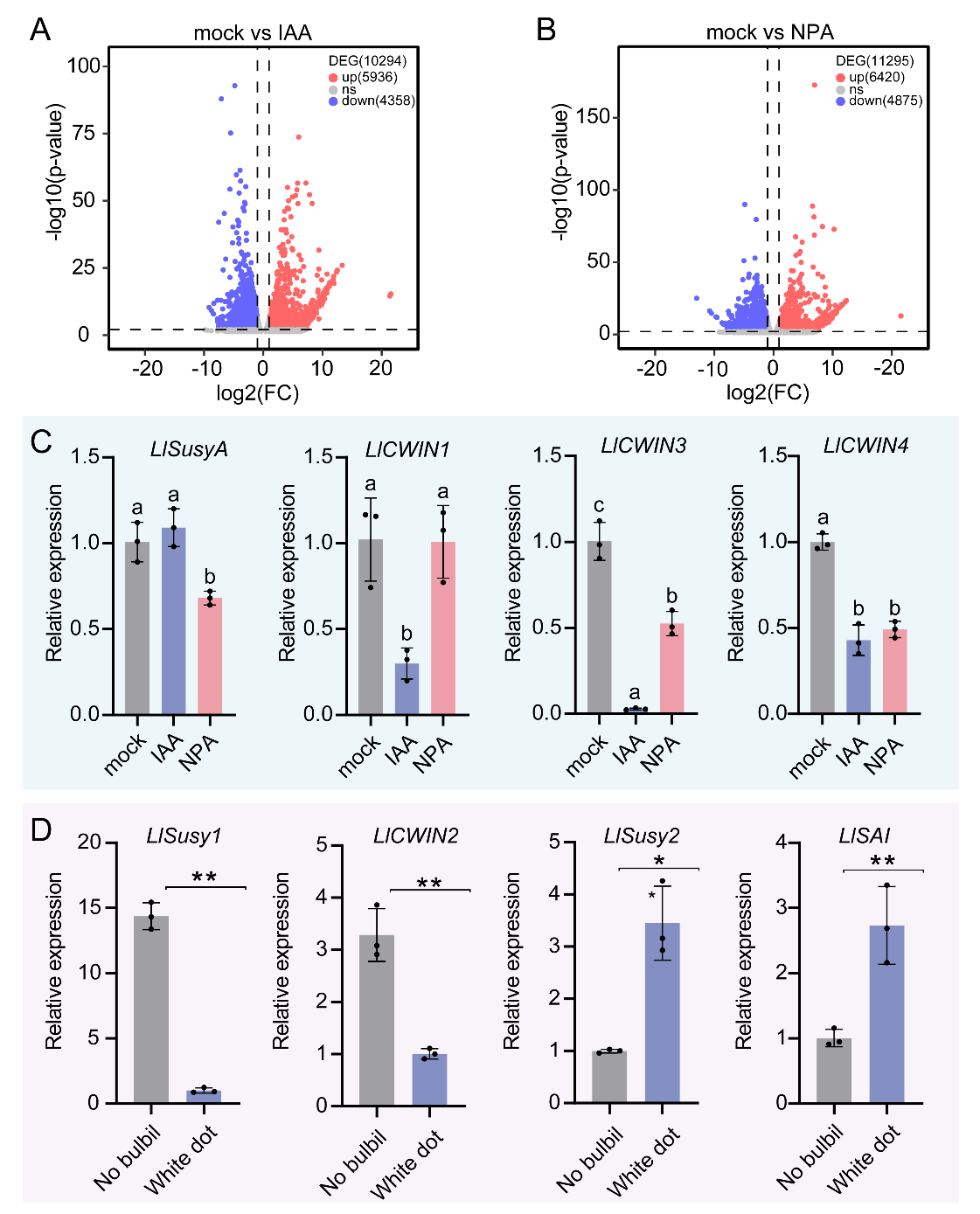


**Supplementary Figure S2. IAA and NPA treatments affect sucrose metabolism.**

**(A-B)** The volcano chart showed the distribution of DEGs in the mock vs. IAA comparison **(A)** and mock vs NPA comparison **(B)**. The dotted line represents the threshold of DEGs screening criteria. The red and blue dots represent up- and down-regulated genes, respectively, and the black dots represent genes with no significant differential expression. **(C)** The expression pattern of *LlSusyA*, *LlCWIN1*, *LlCWIN3*, and *LlCWIN4* of leaf axils in the mock, IAA, and NPA treatments. **(D)** The expression pattern of *LlSusy1*, *LlCWIN2*, *LlSusy2*, and *LlSAI* in leaf axils at ‘No bulbil’ and ‘White dot’ stages. Three biological replicates in panels **C** and **D** were performed. The lowercase letters in panel **C** represented significant differences calculated by an ANOVA and post hoc Tukey’s HSD (P＜0.05). Student’s *t*-test was used for statistical analysis in panel **D** (*: P < 0.05; **: P < 0.01).


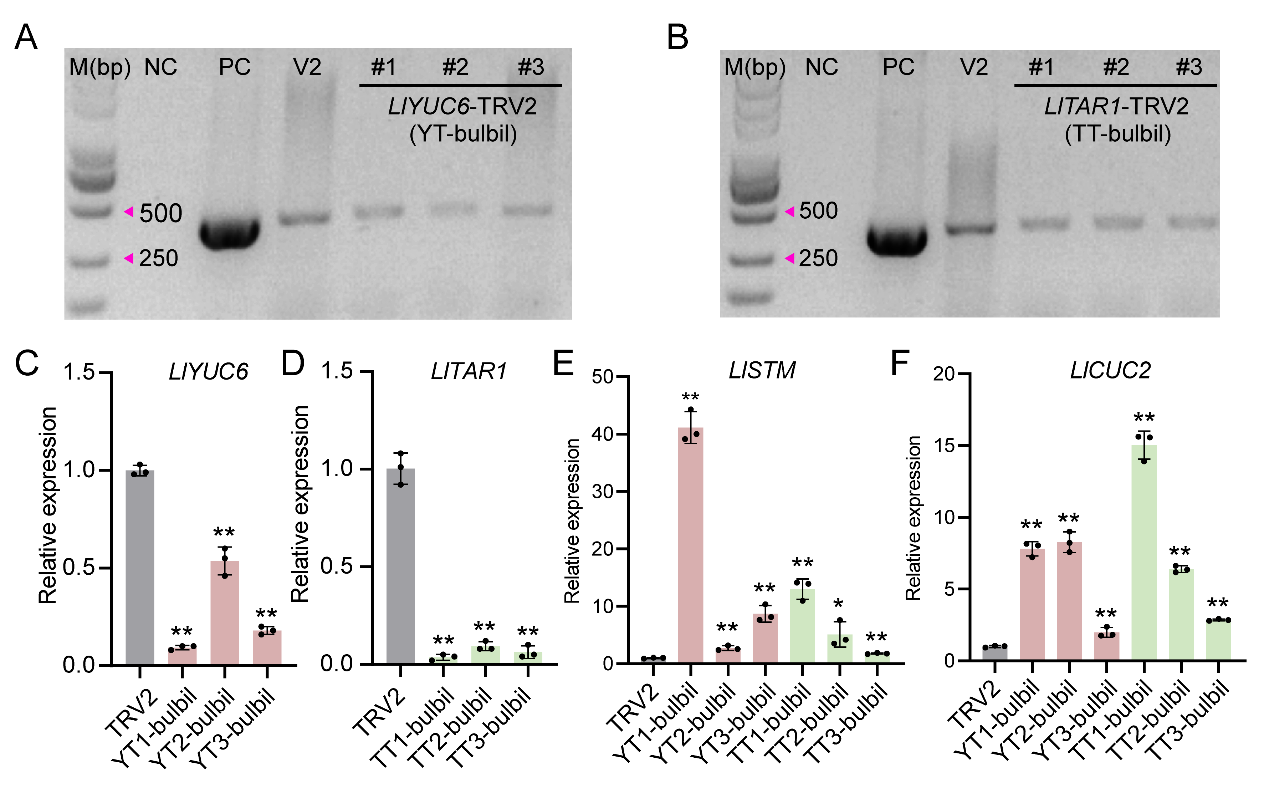


**Supplementary Figure S3. Validation of *LlYUC6-*silenced and *LlTAR1-*silenced plants. (A-B)** Detection of the virus *COAT PROTEIN* fragments in the upper leaf axils of TRV2 (V2), *LlYUC6*-TRV2 (YT-bulbil) **(A)**, and *LlTAR1*-TRV2 (TT-bulbil) **(B)** plants using RT-PCR with extracted RNA. M: DNA marker; NC: non-agroinfiltrated plants used as the negative control; PC: recombinant pTRV2 plasmids with *LlYUC6* specific fragment **(A)** or *LlTAR1* specific fragment **(B)** were used as positive controls. **(C)** Expression of *LlYUC6* in the upper leaf axils of *LlYUC6*-TRV2 and TRV2 control plants. **(D)** Expression of *LlTAR1* in the upper leaf axils of *LlTAR1-*TRV2 and TRV2 control plants. **(E-F)** Expression of *LlSTM* **(E)** and *LlCUC2* **(F)** in the upper leaf axils of *LlYUC6*-TRV2, *LlTAR1-*TRV2, and TRV2 control plants. The expression in panels **C-F** was detected by RT-qPCR and the data represents mean ± s.d. of three biological replicates. Student’s *t*-test was used for statistical analysis (*: P < 0.05; **: P < 0.01).


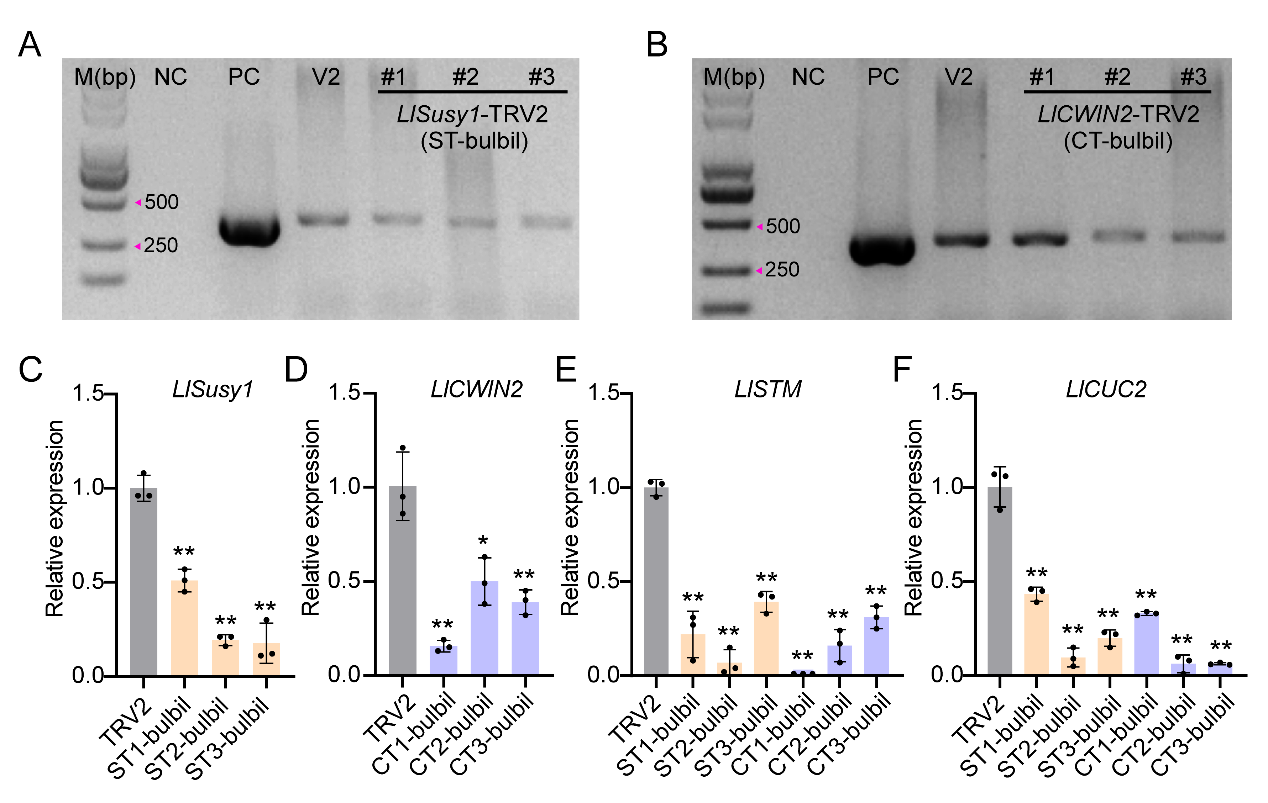


**Supplementary Figure S4. Validation of *LlSusy1-*silenced and *LlCWIN2-*silenced plants.**

**(A-B)** Detection of the virus *COAT PROTEIN* fragments in the upper leaf axils of TRV2 (V2), *LlSusy1-*TRV2 (ST-bulbil) **(A)** and *LlCWIN2*-TRV2 (CT-bulbil) **(B)** plants using RT-PCR with extracted RNA. M: DNA marker; NC: non-agroinfiltrated plants used as the negative control; PC: recombinant pTRV2 plasmids with *LlSusy1* specific fragment **(A)** or *LlCWIN2* specific fragment **(B)** were used as positive controls. **(C)** Expression of *LlSusy1* in the upper leaf axils of *LlSusy1*-TRV2 and TRV2 control plants. **(D)** Expression of *LlCWIN2* in the upper leaf axils of *LlCWIN2-*TRV2 and TRV2 control plants. **(E-F)** Expression of *LlSTM* **(E)** and *LlCUC2* **(F)** in the upper leaf axils of *LlSusy1*-TRV2, *LlCWIN2-*TRV2 and TRV2 control plants. The expression in panels **C-F** was detected by RT-qPCR and the data represents mean ± s.d. of three biological replicates. Student’s *t*-test was used for statistical analysis (*: P < 0.05; **: P < 0.01).

**
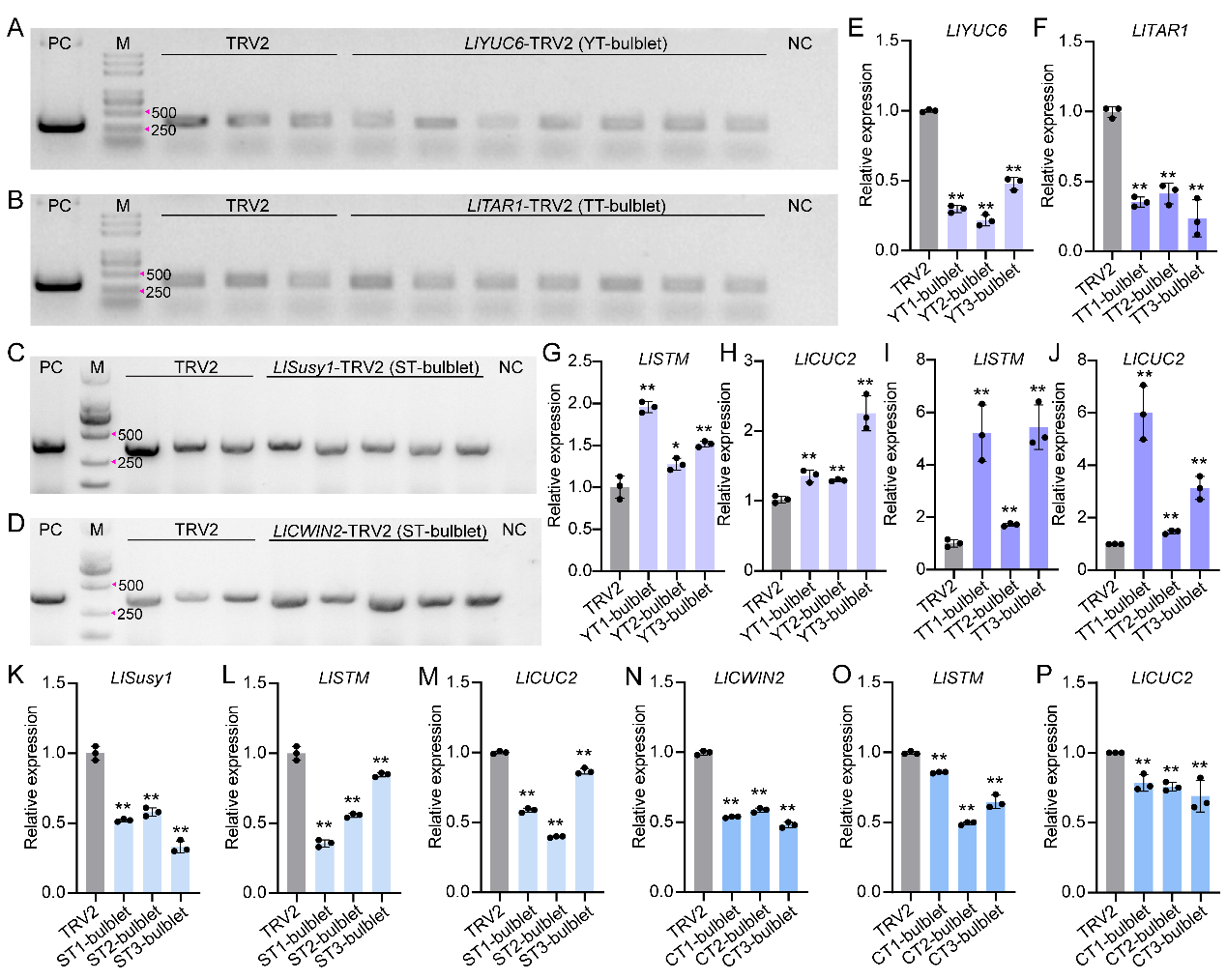
**

**Supplementary Figure S5. Validation of *LlYUC6-*, LlTAR1*-*, *LlSusy1-*, and *LlCWIN2-* silenced scales.**

**(A-D)** Detection of the virus *COAT PROTEIN* fragments in the base of TRV2, *LlYUC6*-TRV2 (YT-bulblet) **(A)**, *LlTAR1*-TRV2 (TT-bulblet) **(B)**, *LlSusy1-*TRV2 (ST-bulblet) **(C)**, and *LlCWIN2*-TRV2 (CT-bulblet) **(D)** scales using RT-PCR with extracted RNA. M: DNA marker (bp); NC: non-agroinfiltrated plants used as the negative control; PC: recombinant pTRV2 plasmids with the specific fragment *of LlYUC6* **(A)**, *LlTAR1* **(B)**, *LlSusy1* **(C)**, or *LlCWIN2* **(D)** were used as positive controls. **(E)** Expression of *LlYUC6* in the base of *LlYUC6*-TRV2 and TRV2 control scales. **(F)** Expression of *LlTAR1* in the base of *LlTAR1*-TRV2 and TRV2 control scales. **(G-H)** Expression of *LlSTM* **(G)** and *LlCUC2* **(H)** in the base of *LlYUC6*-TRV2 and TRV2 control scales. **(I-J)** Expression of *LlSTM* **(I)** and *LlCUC2* **(J)** in the base of *LlTAR1*-TRV2 and TRV2 control scales. **(K)** Expression of *LlSusy1* in the base of *LlSusy1*-TRV2 and TRV2 control scales. **(L-M)** Expression of *LlSTM* **(L)** and *LlCUC2* **(M)** in the base of *LlSusy1*-TRV2 and TRV2 control scales. **(N)** Expression of *LlCWIN2* in the base of *LlCWIN2*-TRV2 and TRV2 control scales. **(O-P)** Expression of *LlSTM* **(O)** and *LlCUC2* **(P)** in the base of *LlCWIN2*-TRV2 and TRV2 control scales. The expression in panels **E-P** was detected by RT-qPCR and the data represents mean ± s.d. of three biological replicates. Student’s *t*-test was used for statistical analysis (*: P < 0.05; **: P < 0.01).


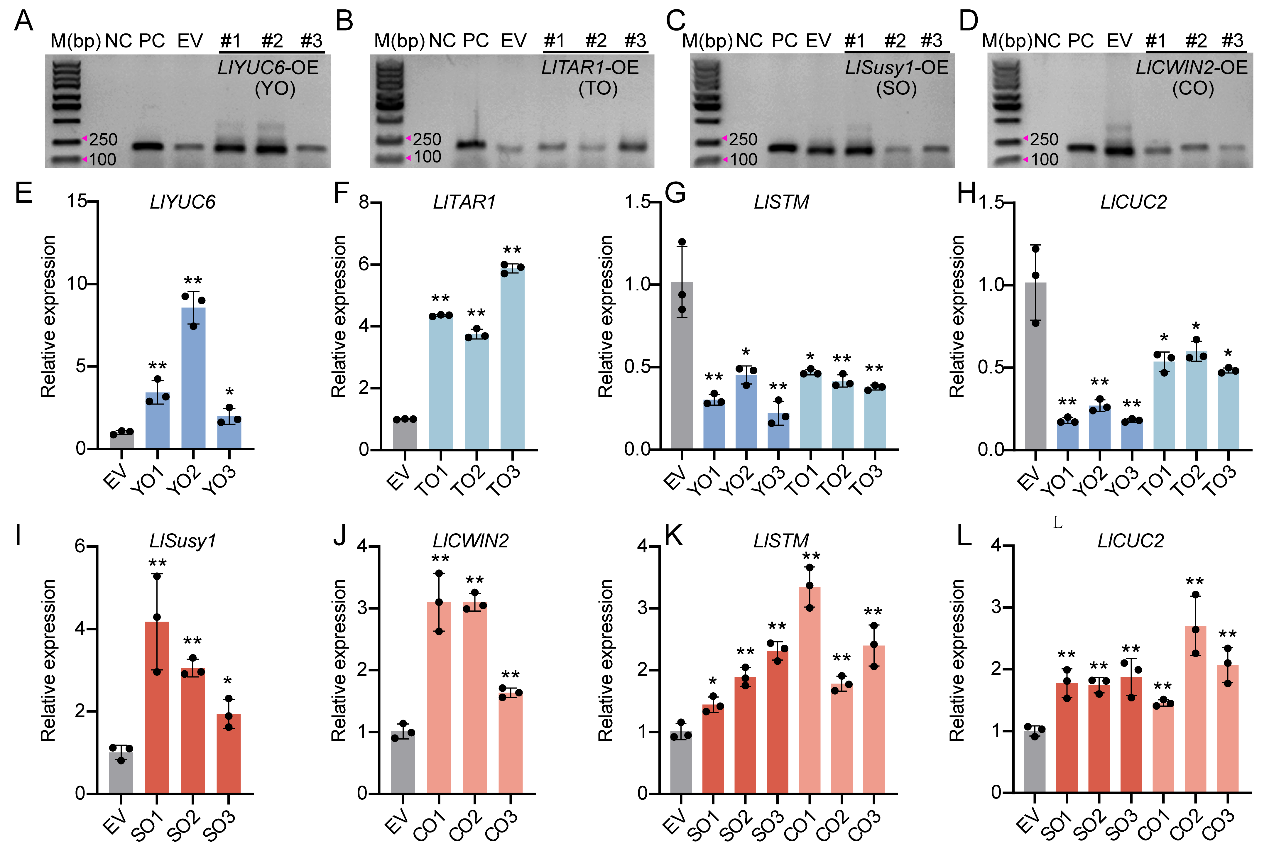


**Supplementary Figure S6. Validation of *LlYUC6-*, LlTAR1*- LlSusy1-* and *LlCWIN2-* overexpressed scales.**

**(A)-(D)** Detection of *eGFP* fragment (reporter gene) in EV (35S: eGFP; empty vector) control, *LlYUC6*-OE (YO) **(A)**, *LlTAR1*-OE (TO) **(B)**, *LlSusy1*-OE (SO) **(C)**, and *LlCWIN2*-OE (CO) **(D)** scales using RT-PCR with extracted RNA. M: DNA marker; NC: non-agroinfiltrated scales used as the negative control; PC: recombinant pCAMBIA2300 plasmids with 35S: eGFP-*LlYUC6* **(A)**, 35S: eGFP-*LlTAR1* **(B)**, 35S: eGFP-*LlSusy1* **(C)**, or 35S: eGFP-*LlCWIN2* **(D)** were used as positive controls. **(E)** Expression of *LlYUC6* in the base of *LlYUC6*-OE and EV (empty vector; 35S: eGFP) scales. **(F)** Expression of *LlTAR1* in the base of *LlTAR1*-OE and EV scales. **(G-H)** Expression of *LlSTM* **(G)** and *LlCUC2* **(H)** in the base of *LlYUC6*-OE, *LlTAR1*-OE, and EV control scales. **(I)** Expression of *LlSusy1* in the base of *LlSusy1*-OE and EV control scales. **(J)** Expression of *LlCWIN2* in the base of *LlCWIN2*-OE and EV control scales. **(K-L)** Expression of *LlSTM* **(K)** and *LlCUC2* **(L)** in the base of *LlSusy1*-OE, *LlCWIN2*-OE and EV control scales. The expression in panels **E-L** was detected by RT-qPCR and the data represents mean ± s.d. of three biological replicates. Student’s *t*-test was used for statistical analysis (*: P < 0.05; **: P < 0.01).

**
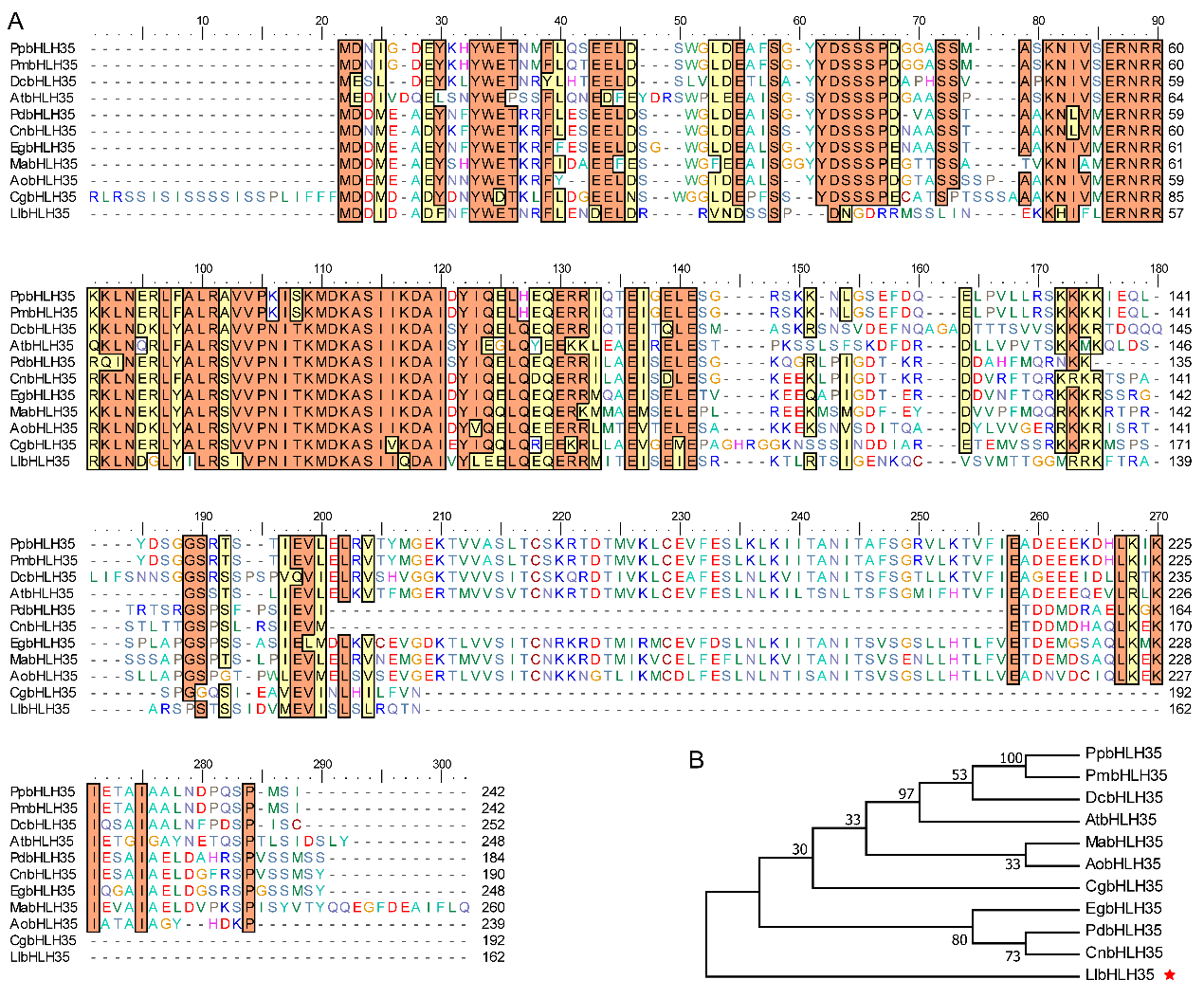
**

**Supplementary Figure S7. Amino acid sequence alignment and phylogenetic analysis of LlbHLH35.**

**(A)** Protein sequence alignment of LlbHLH35 proteins and its homologs. **(B)** Phylogenetic tree analysis of LlbHLH35 and its homologs. AtbHLH35 (Q2HIV9.1) in *Arabidopsis thaliana*, AobHLH35 (XP_020267843.1) in *Asparagus officinalis*, CnbHLH35 (KAG1331957.1) in *Cocos nucifera*, CgbHLH35 (QDF82451.1) in *Cymbidium goeringii*, DcbHLH35 (XP_017250303.1) in *Daucus carota*, EgbHLH35 (XP_010929341.1) in *Elaeis guineensis*, MabHLH35 (XP_009409750.1) in *Musa acuminata*, PdbHLH35 (XP_017699987.1) in *Phoenix dactylifera*, PmbHLH35 (XP_008223870.1) in *Prunus mume* and PpbHLH35 (XP_007223913.2) in *Prunus persica* were used.

**
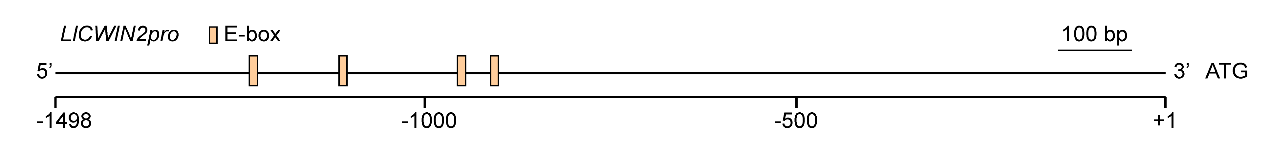
**

**Supplementary Figure S8. Schematic diagram of *LlCWIN2* genomic regions upstream of the start codon.** The yellow box represented E-box element. Thin black lines bar represented 100 bp.


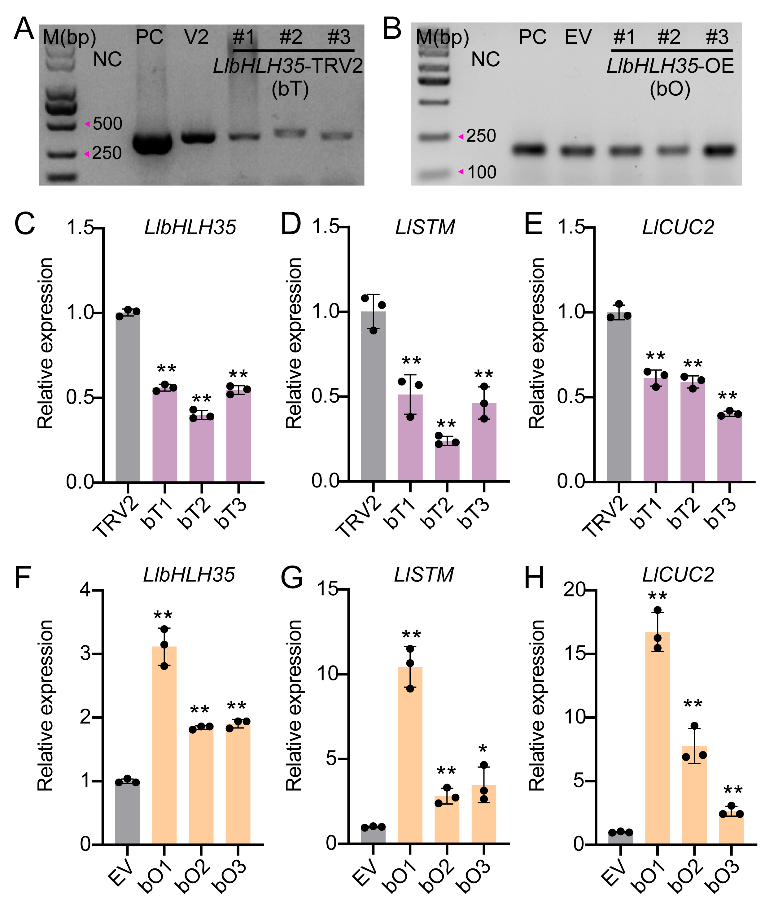


**Supplementary Figure S9. Validation of *LlbHLH35*-silenced and *LlbHLH35-*overexpressed scales.**

**(A)** Detection of the virus *COAT PROTEIN* fragments in the base of TRV2 (V2) and *LlbLH35*-TRV2 (bT) plants using RT-PCR with extracted RNA. M: DNA marker; NC: non-agroinfiltrated plants used as the negative control; PC: recombinant pTRV2 plasmids with *LlbHLH35* specific fragment were used as positive controls. **(B)** Detection of *eGFP* fragment (reporter gene) in EV (empty vector; 35S: eGFP) control and *LlbHLH35*-OE (bO) scales using RT-PCR with extracted RNA. M: DNA marker; NC: non-agroinfiltrated scales used as the negative control; PC: recombinant pCAMBIA2300 plasmids with 35S: eGFP-*LlbHLH35* were used as positive controls. **(C)** Expression of *LlbHLH35* in the base of *LlbHLH35*-TRV2 and TRV2 control scales. **(D-E)** Expression of *LlSTM* **(D)** *and LlCUC2* **(E)** in the base of *LlbHLH35*-TRV2 and TRV2 control scales. **(F)** Expression of *LlbHLH35* in the base of *LlbHLH35*-OE and EV control scales. **(G-H)** Expression of *LlSTM* **(G)** *and LlCUC2* **(H)** in the base of *LlbHLH35*-OE and EV control scales. The expression in panels **C-H** was detected by RT-qPCR and the data represents mean ± s.d. of three biological replicates. Student’s *t*-test was used for statistical analysis (*: P < 0.05; **: P < 0.01).

**
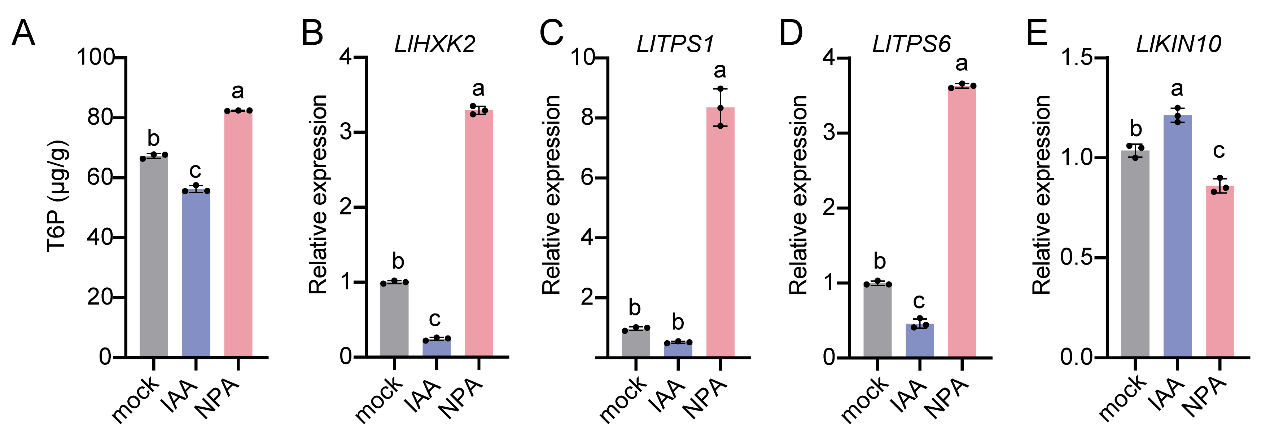
**

**Supplementary Figure S10. NPA and IAA treatments affect T6P content. (A)** The content of T6P of upper leaf axils in the mock, IAA, and NPA treatments. Three biological replicates were performed. **(B-E)** Expression of sugar-related genes, *LlHXK2* **(B)**, *LlTPS1* **(C)**, *LlTPS6* **(D)**, and *LlKIN10* **(E)** in the upper leaf axils of mock, IAA, and NPA treatments. The expression in panels **B-E** was detected by RT-qPCR and the data represents mean ± s.d. of three biological replicates. The lowercase letters in panels **A-E** represent significant differences calculated by an ANOVA and post hoc Tukey’s HSD (P＜0.05).
