# Supplementary figures and images for: Auxin regulates bulbil initiation by mediating sucrose metabolism in Lilium lancifolium
Source: Hortic Res. 2024 Feb 23;11(4):uhae054. doi: 10.1093/hr/uhae054 (PMC11069426; doi:10.1093/hr/uhae054)

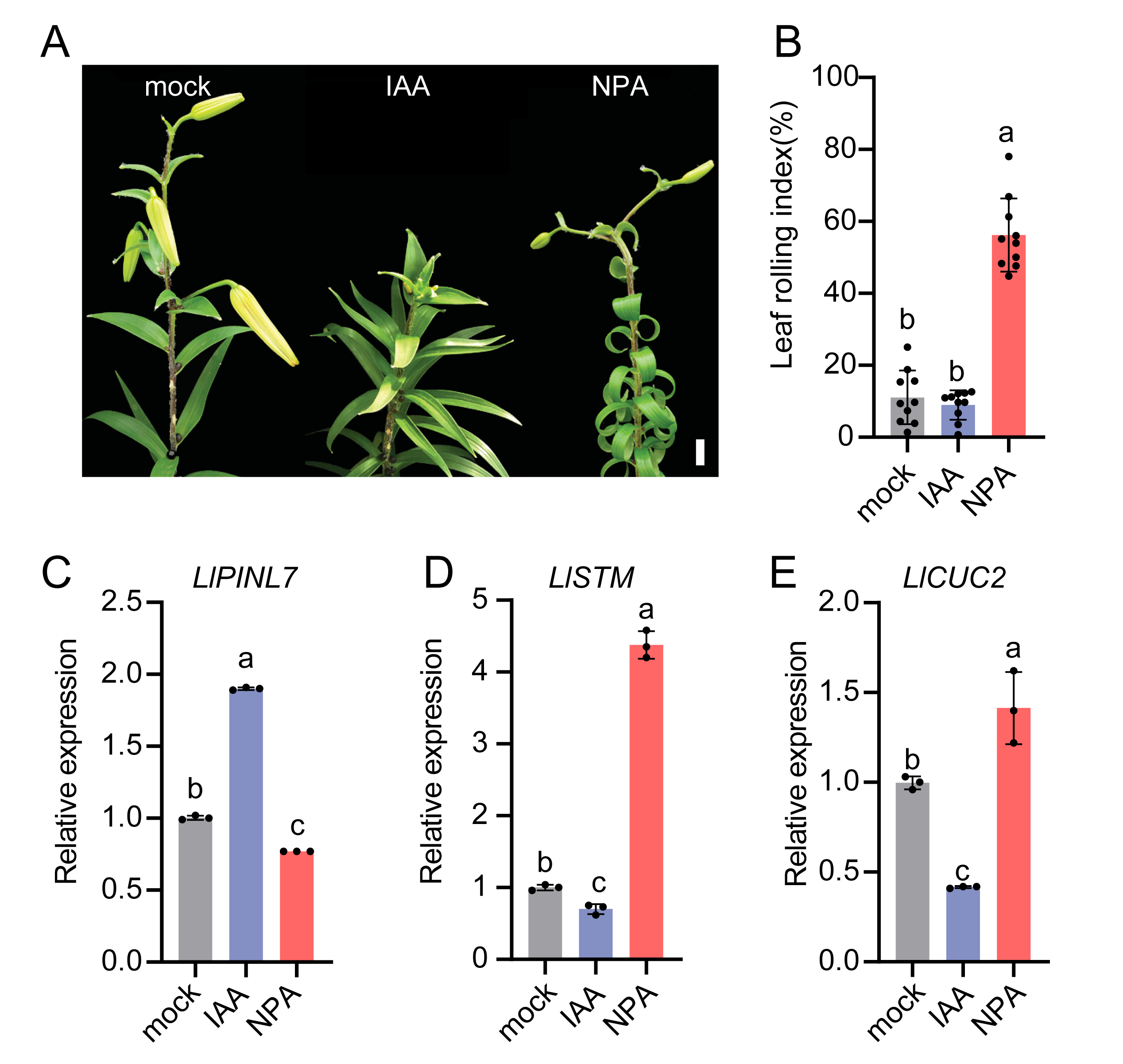

Supplement: Web_Material_uhae054 [file web_material_uhae054.zip › Supplementary Figure S1.tif]

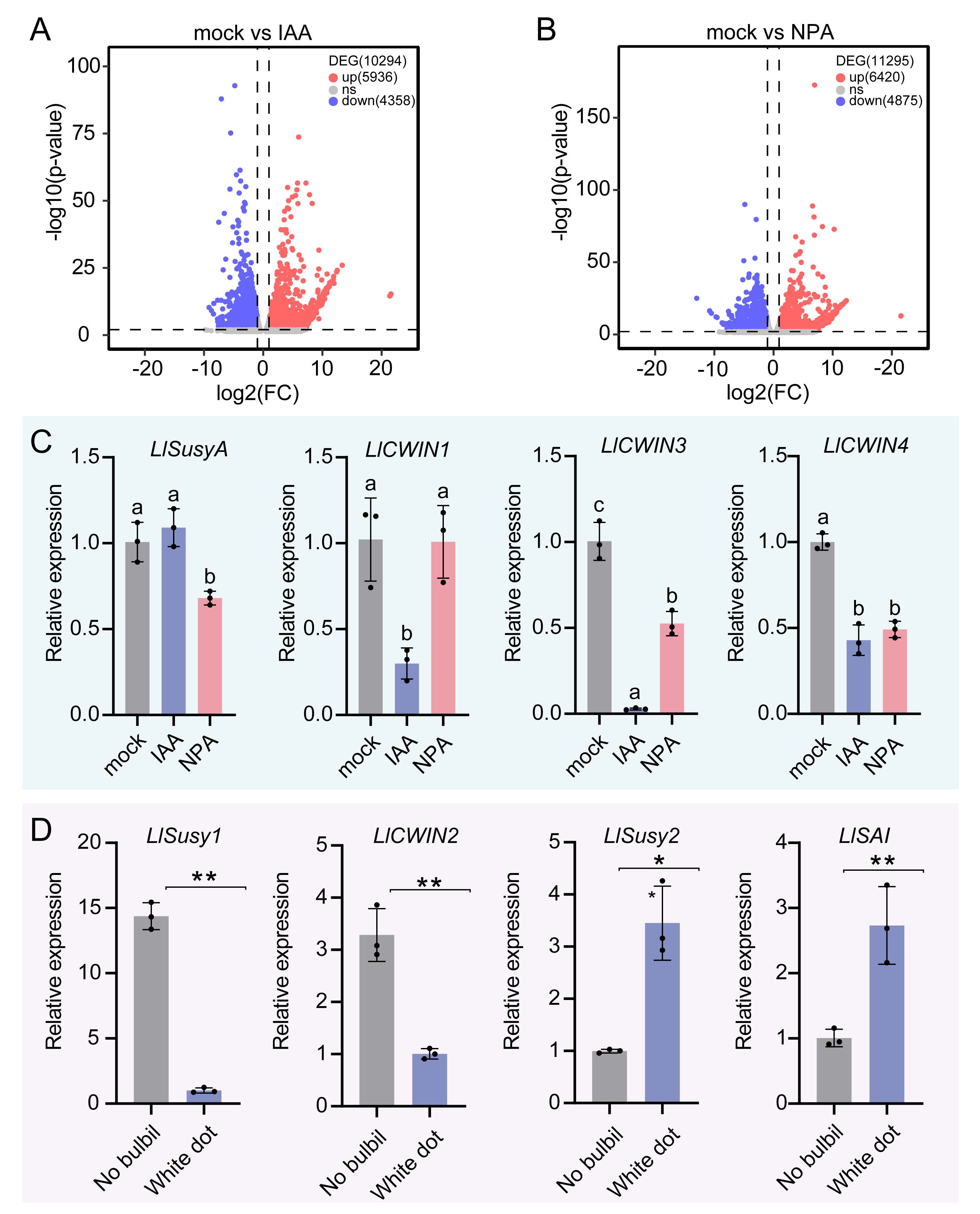

Supplement: Web_Material_uhae054 [file web_material_uhae054.zip › Supplementary Figure S2.tif]

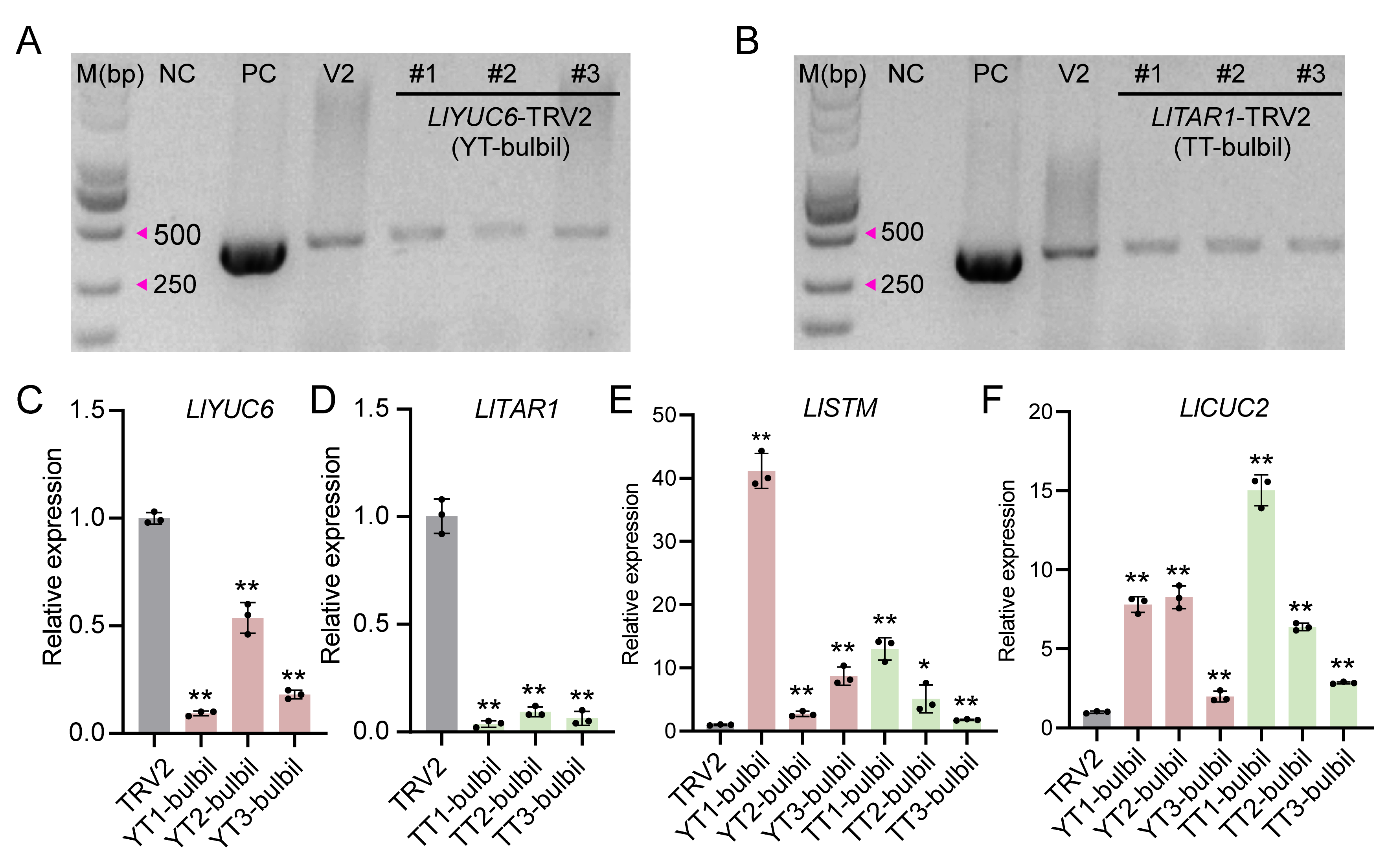

Supplement: Web_Material_uhae054 [file web_material_uhae054.zip › Supplementary Figure S3.tif]

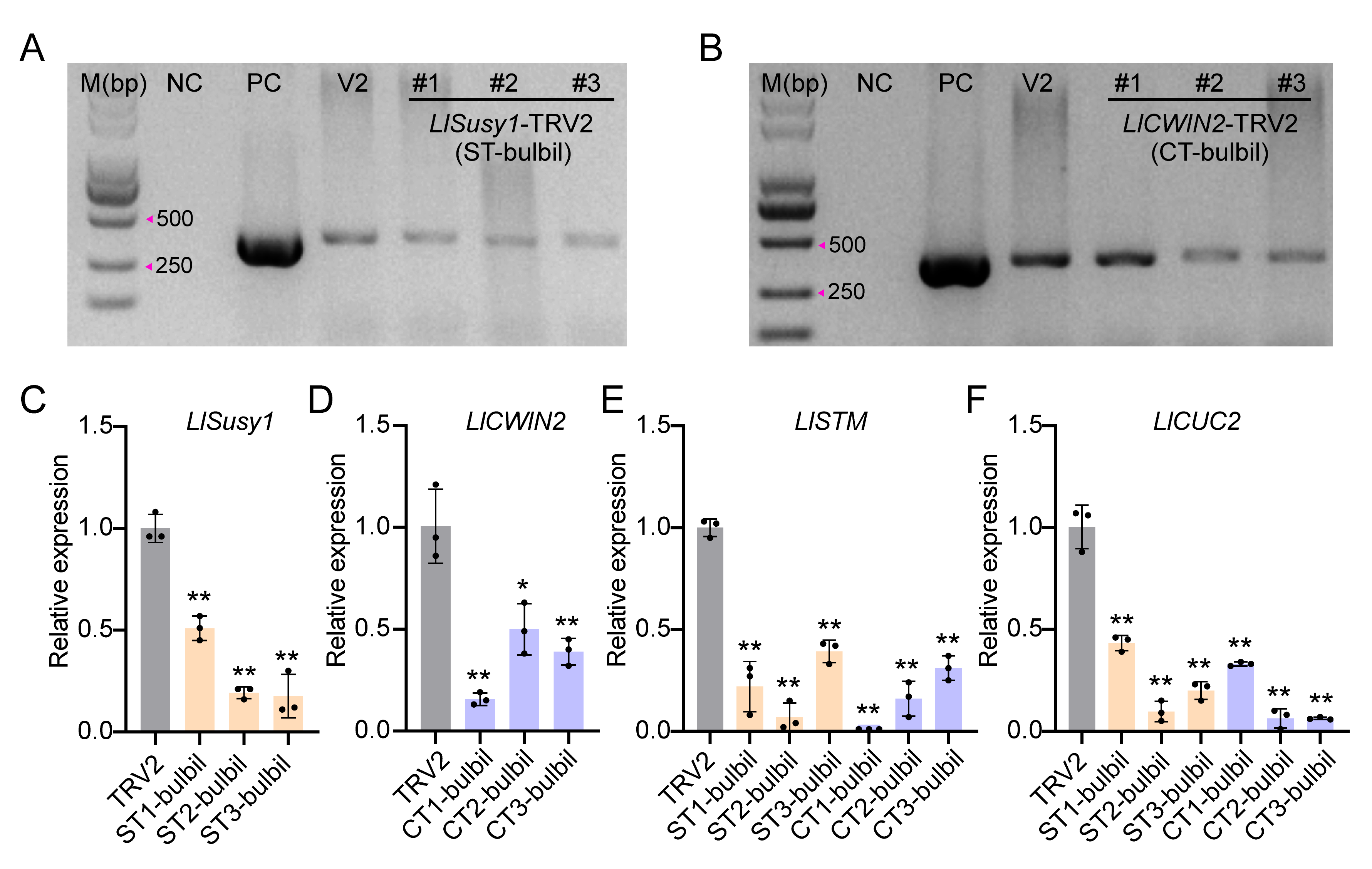

Supplement: Web_Material_uhae054 [file web_material_uhae054.zip › Supplementary Figure S4.tif]

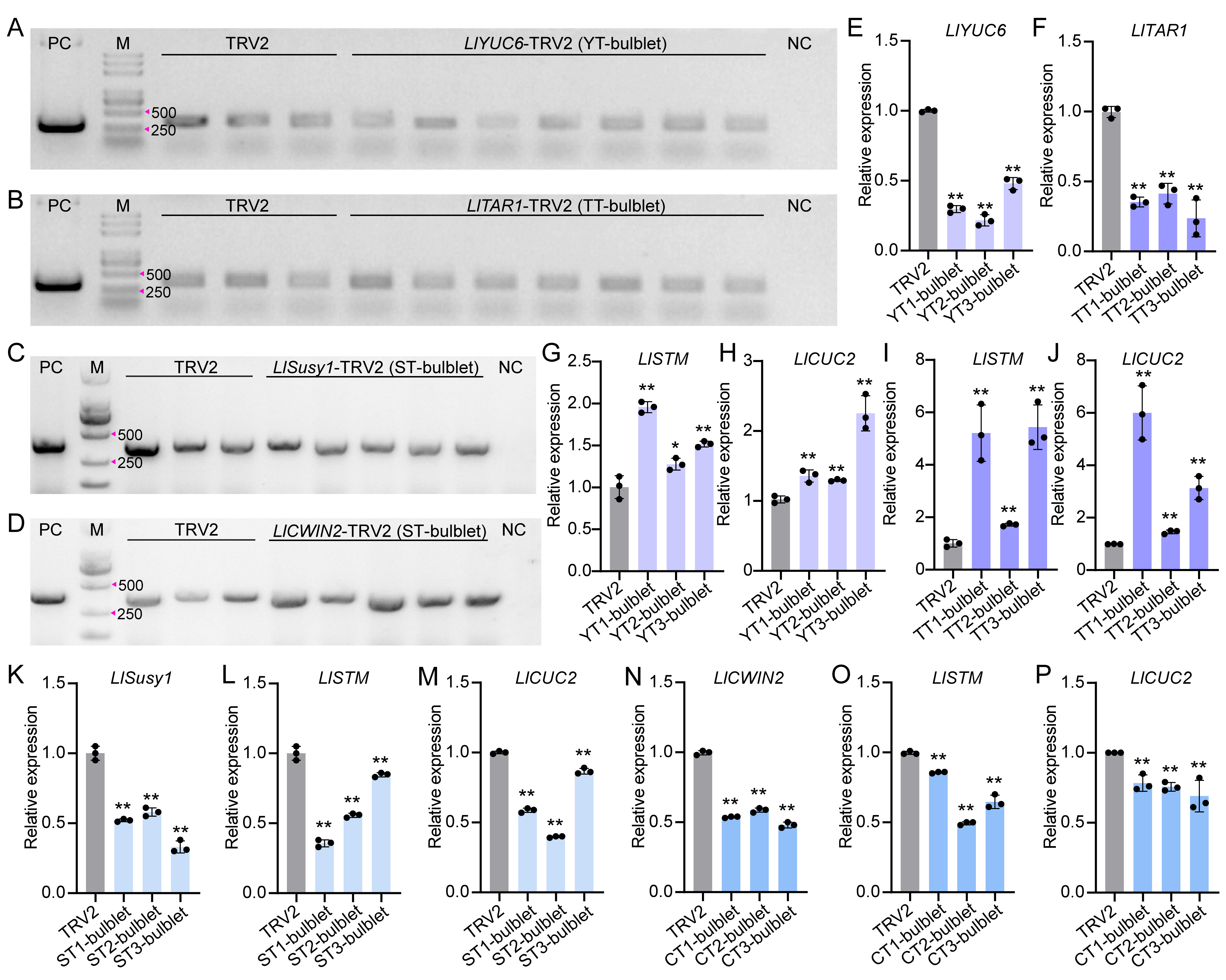

Supplement: Web_Material_uhae054 [file web_material_uhae054.zip › Supplementary Figure S5.tif]

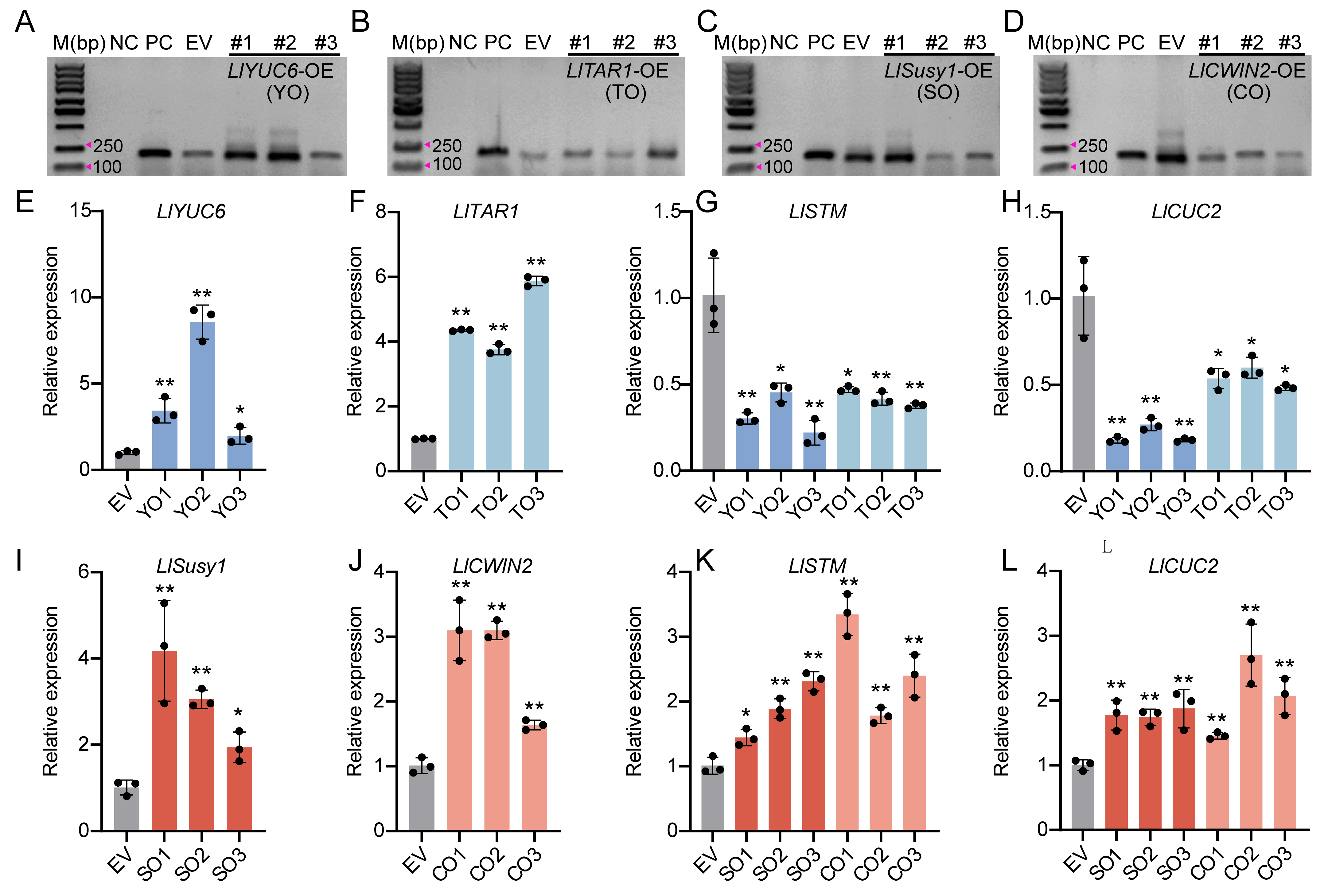

Supplement: Web_Material_uhae054 [file web_material_uhae054.zip › Supplementary Figure S6.tif]

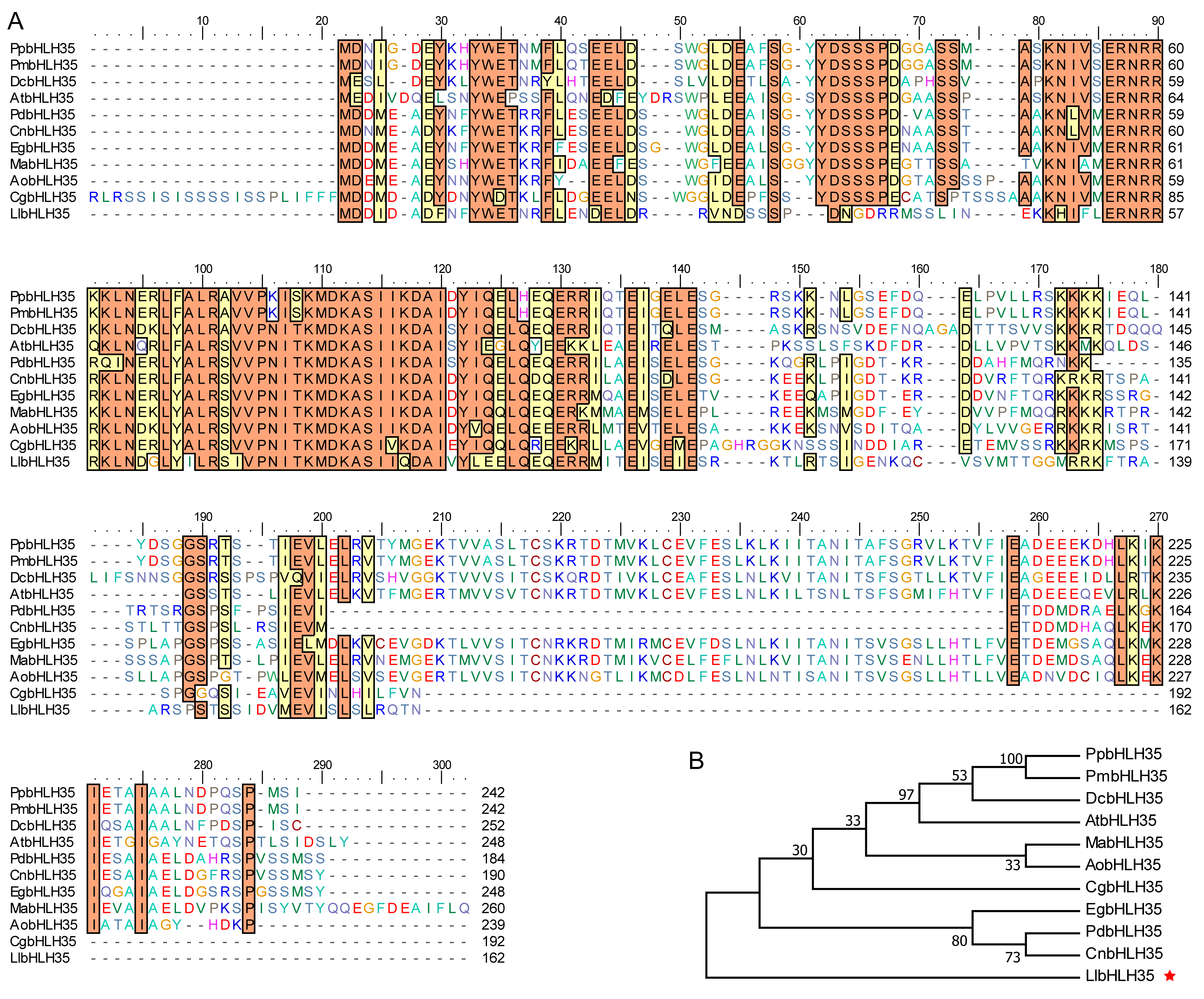

Supplement: Web_Material_uhae054 [file web_material_uhae054.zip › Supplementary Figure S7.tif]

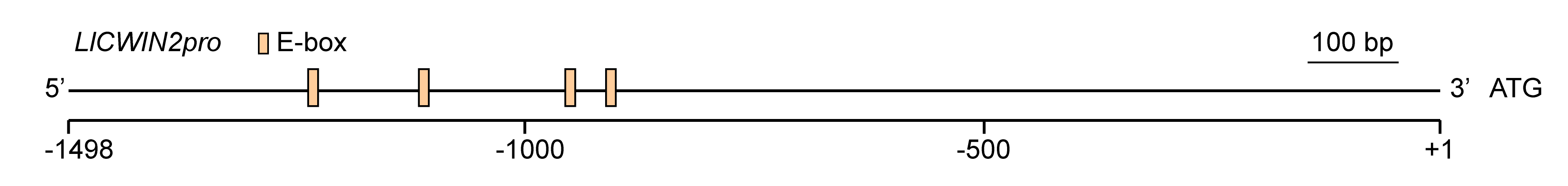

Supplement: Web_Material_uhae054 [file web_material_uhae054.zip › Supplementary Figure S8.tif]

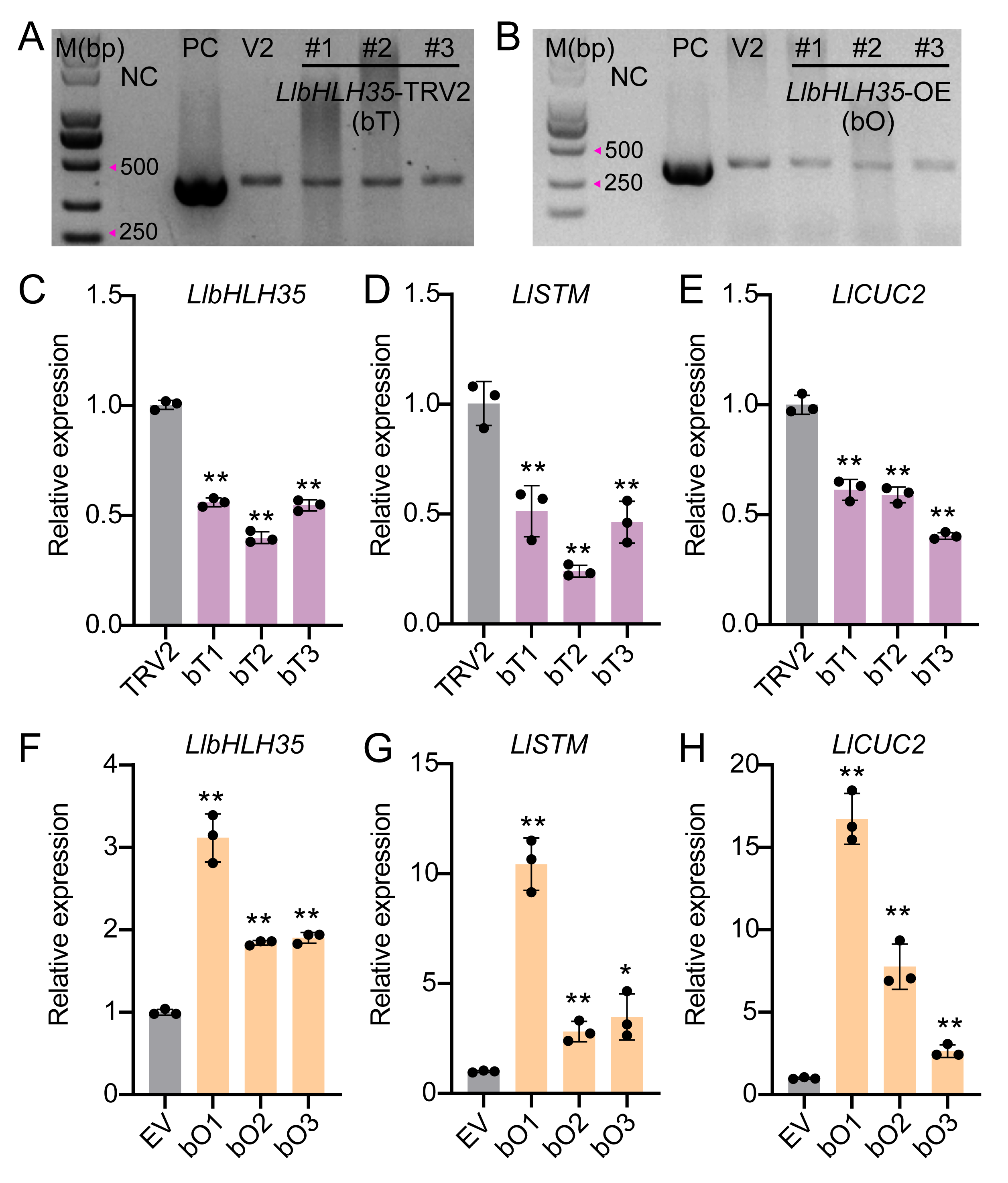

Supplement: Web_Material_uhae054 [file web_material_uhae054.zip › Supplementary Figure S9.tif]

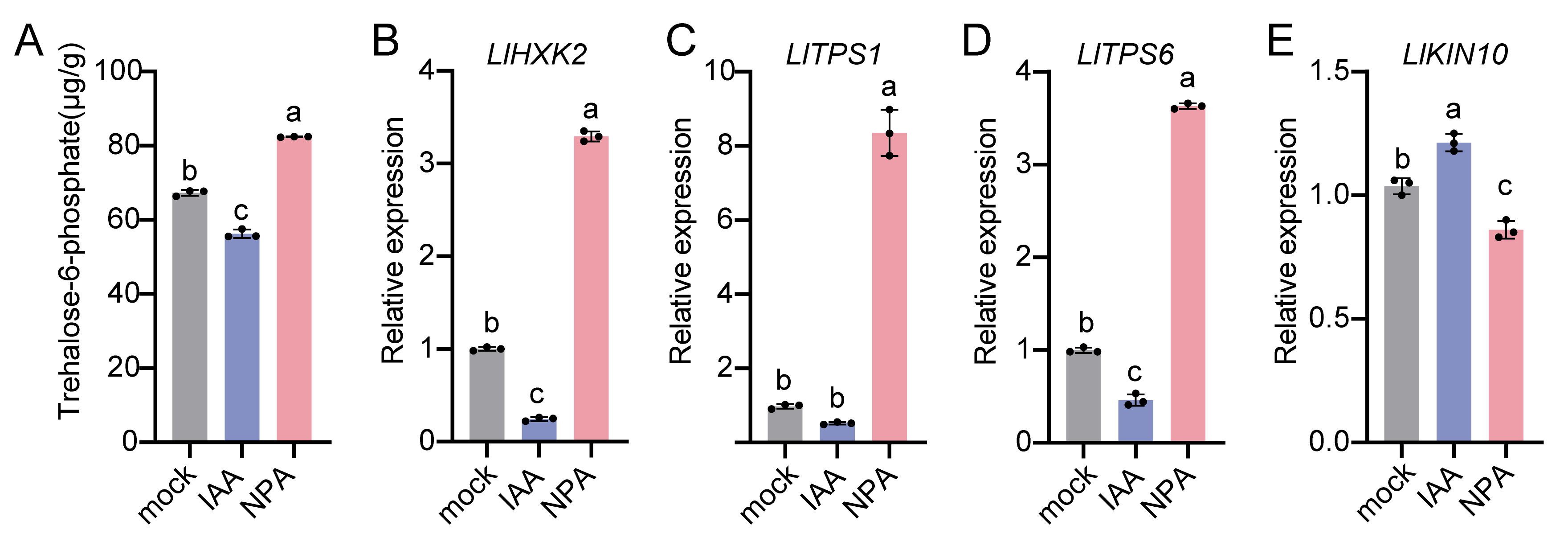

Supplement: Web_Material_uhae054 [file web_material_uhae054.zip › Supplementary Figure S10.tif]
